# Supplementary material for: Probing the binding of interleukin-23 to individual receptor components and the IL-23 heteromeric receptor complex in living cells using NanoBRET
Source: Cell Chem Biol. 2022 Jan 20;29(1):19–29.e6. doi: 10.1016/j.chembiol.2021.05.002 (PMC8790524; doi:10.1016/j.chembiol.2021.05.002)
Supplement: Document S1. Figures S1–S6 and Table S1 [file mmc1.pdf]

Cell Chemical Biology, Volume *29*

Supplemental information

Probing the binding of interleukin-23 to individual receptor components and the IL-23 heteromeric receptor complex in living cells using NanoBRET

Charles S. Lay, Angela Bridges, Joelle Goulding, Stephen J. Briddon, Zoja Soloviev, Peter D. Craggs, and Stephen J. Hill

Protein name	Predicted MW (Da)	Observed mass (Da)	Suspected PTMs
IL23p19-HisTag	19499.8	19499	none
IL12p40	34696.6	36555 (most abundant)	Mannose N-linked glycosylation
IL-23 complex	54181.2	56050 (most abundant)	Mannose N-linked glycosylation

Table S1: The masses of recombinant purified IL-23 subunits and heterodimeric complex used in the study. Predicted molecular weights were generated from the amino acid sequences of the proteins, assuming the reduction of internal disulphide bridges for the cytokine subunits. PTM, post-transaltional modification.

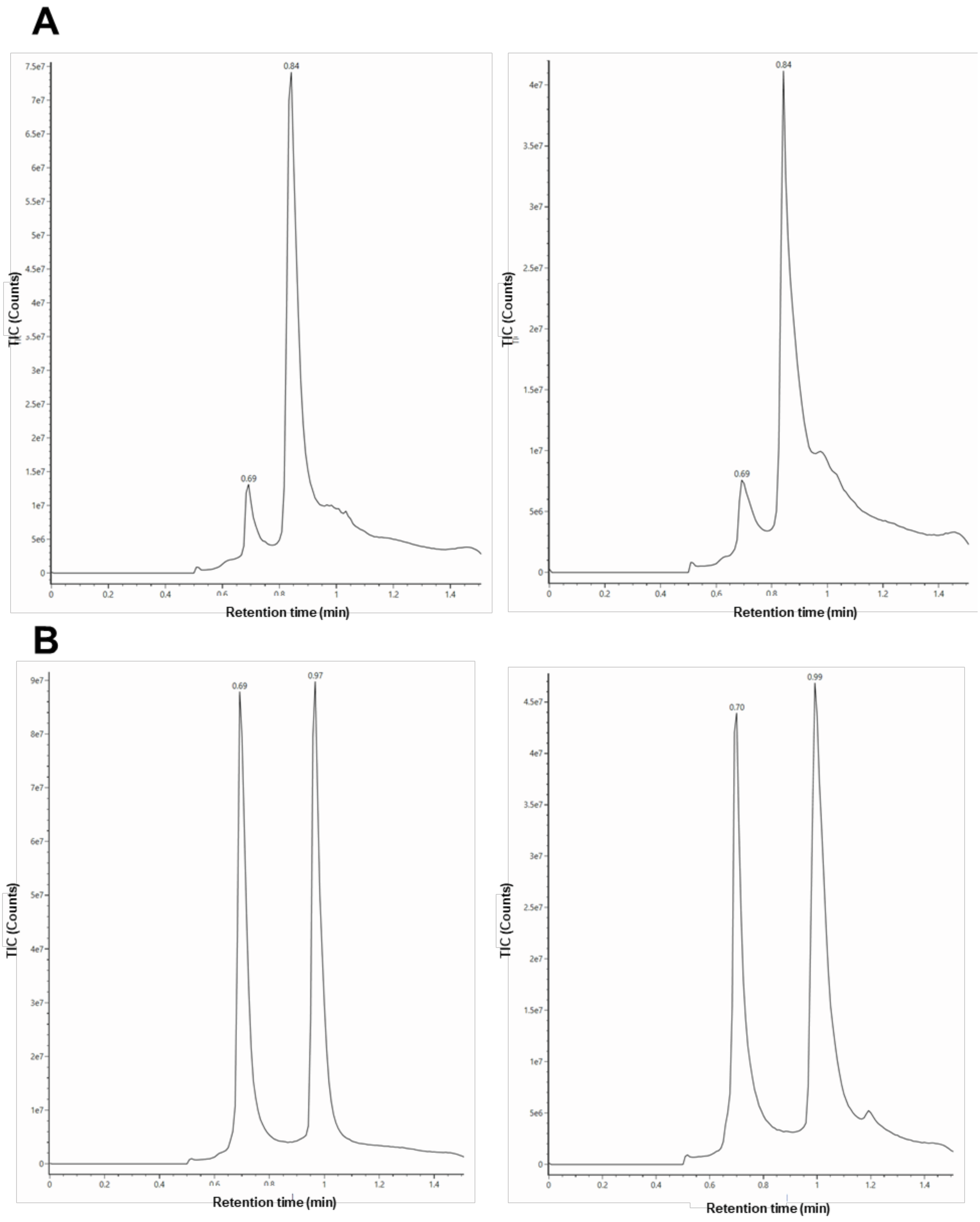

Figure S1 (Related to Figure 1): Reversed-Phase chromatography demonstrates heterodimeric IL-23 is reduced to its subunits by addition of DTT. (A) Total Ion Count (TIC) chromatograms of IL-23 (left) and IL23-TMR (right). (B) Total ion count chromatograms of IL-23 (left) and IL23-TMR (right), in the presence of 50 mM DTT.

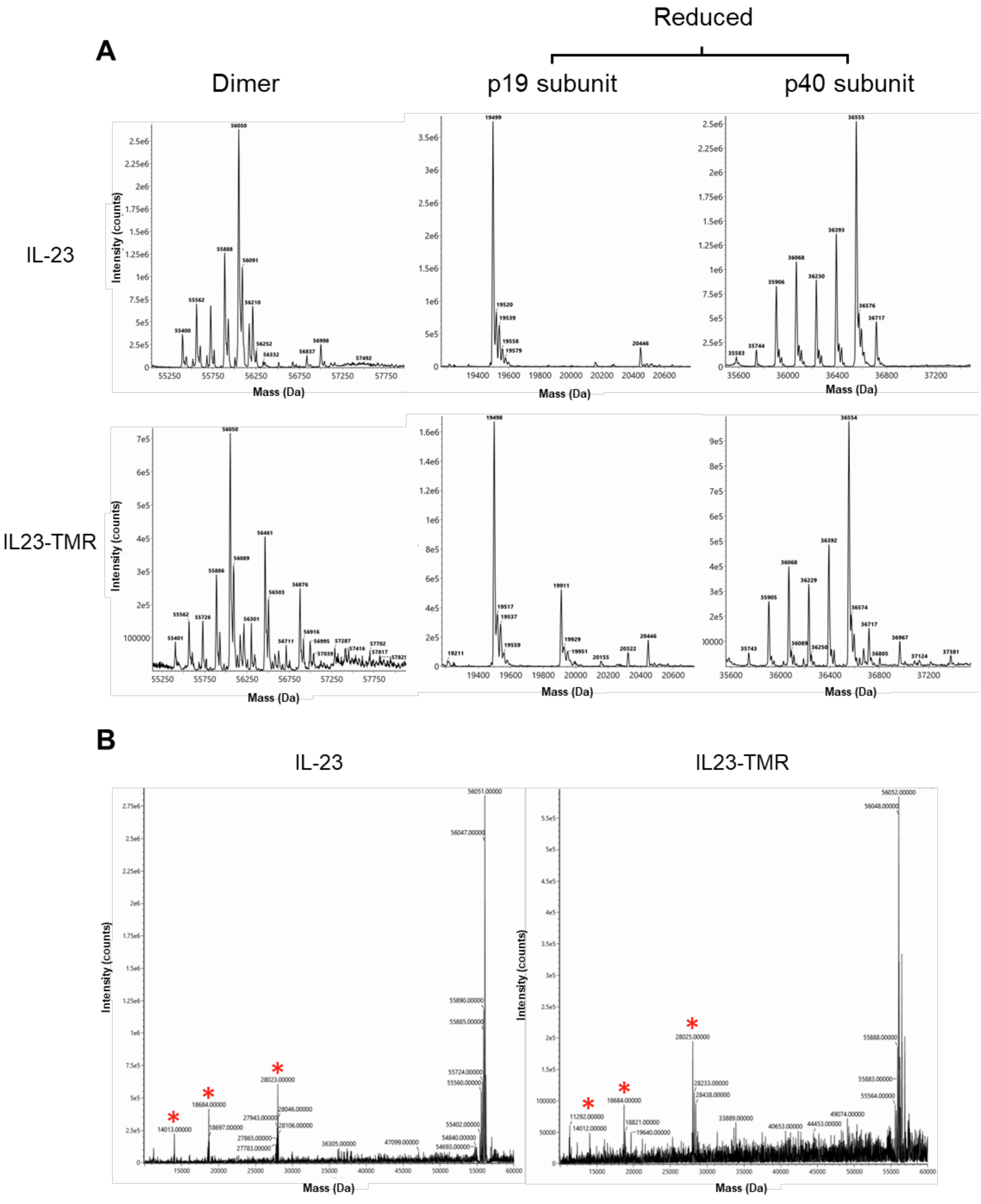

A
Reduced
Dimer
p19 subunit
p40 subunit
IL-23
IL23-TMR
Intensity (counts)
Mass (Da)
56050
55888
56091
55562
56210
55400
56252
56332
56998
56837
57492
19499
19520
19539
19558
19579
20446
36555
36393
36068
36230
35906
36576
36717
35583
35744
56461
55886
56089
55562
55726
55401
56301
56503
56876
56916
56711
56995
57039
57287
57416
57702
57817
19498
19517
19537
19559
19211
19911
19929
19951
20155
20322
36554
36392
36068
36229
35905
36574
36717
36967
35743
36089
36250
36805
37124
37381
B
IL-23
IL23-TMR
56051.00000
56047.00000
55890.00000
55885.00000
55724.00000
55560.00000
55402.00000
54840.00000
54693.00000
28023.00000
28046.00000
27943.00000
28106.00000
27865.00000
27783.00000
18684.00000
18697.00000
14013.00000
36305.00000
47099.00000
56052.00000
56048.00000
55888.00000
55883.00000
55564.00000
28025.00000
28233.00000
28438.00000
18684.00000
18821.00000
19640.00000
11292.00000
14012.00000
33889.00000
40653.00000
44453.00000
49074.00000

Figure S2 (Related to Figure 1): LC-MS analysis of IL23-TMR. (A) De-convolved LC-MS spectra of IL-23 (top) and IL23-TMR (bottom), demonstrating both the DTT reduced, p19 and p40 subunits (centre and right respectively) and the non-reduced dimeric cytokine (left). (B) De-convolved LC-MS spectra of IL-23 (left) and IL23-TMR (right) over a broad mass range. Resonance peak, deconvolution artefacts representing a half, third or fourth the mass of the true species are denoted by a red asterisk.

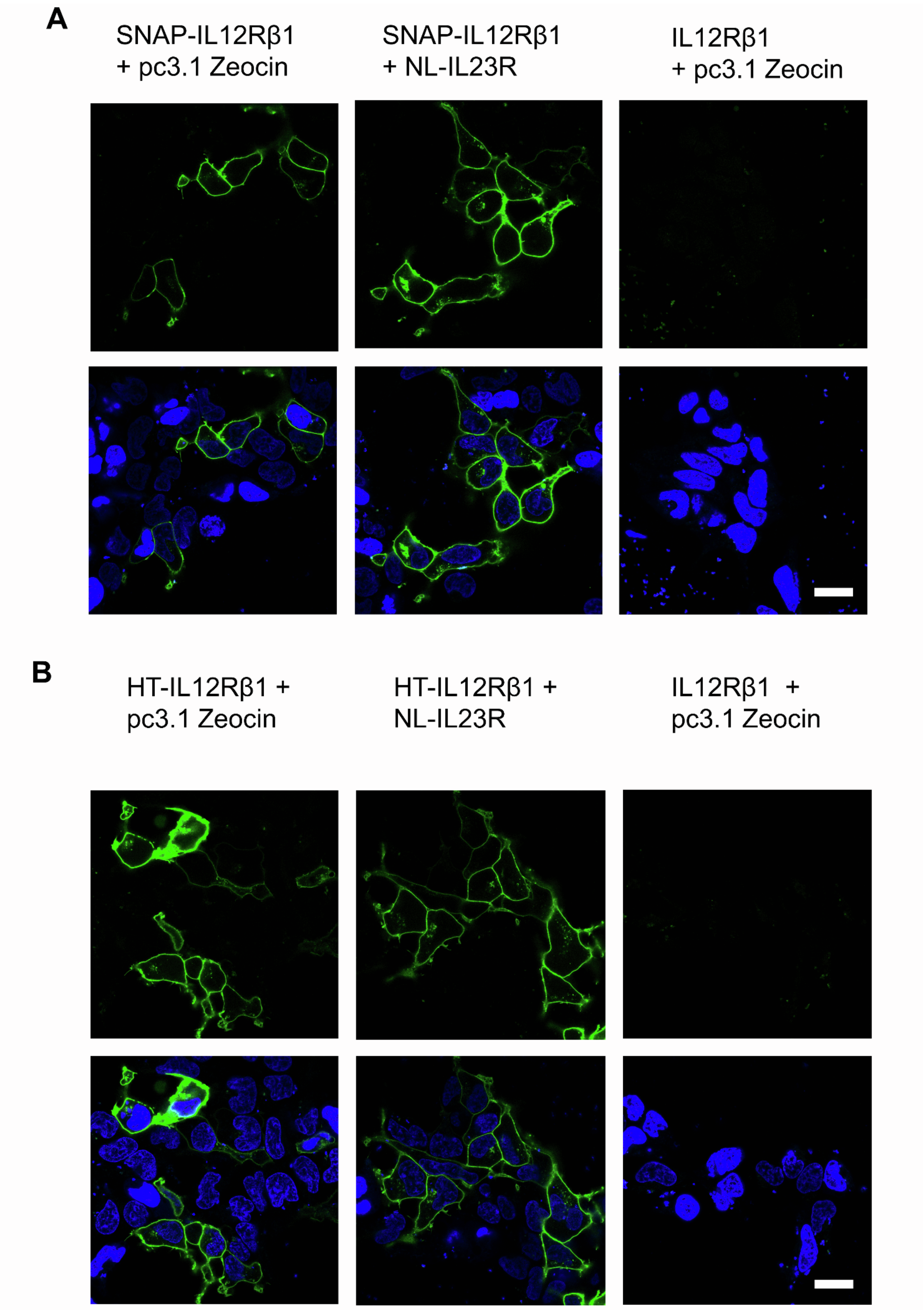
A
SNAP-IL12Rβ1
+ pc3.1 Zeocin
SNAP-IL12Rβ1
+ NL-IL23R
IL12Rβ1
+ pc3.1 Zeocin
B
HT-IL12Rβ1 +
pc3.1 Zeocin
HT-IL12Rβ1 +
NL-IL23R
IL12Rβ1 +
pc3.1 Zeocin

Figure S3 (Related to Figure 2): Surface expression of HaloTag and SNAP-Tag fused IL12Rβ1 constructs. (A) Transfected HEK293T cells labelled with SNAPTag-AF488 substrate with (bottom) and without (top) Hoerscht stain. Representative of 3 independent experiments. (B) Transfected HEK293T cells labelled with AF488 HaloTag Ligand with (bottom) and without (top) Hoerscht stain. Scale bars represent 20 μm. Images are representative of 3 independent experiments.

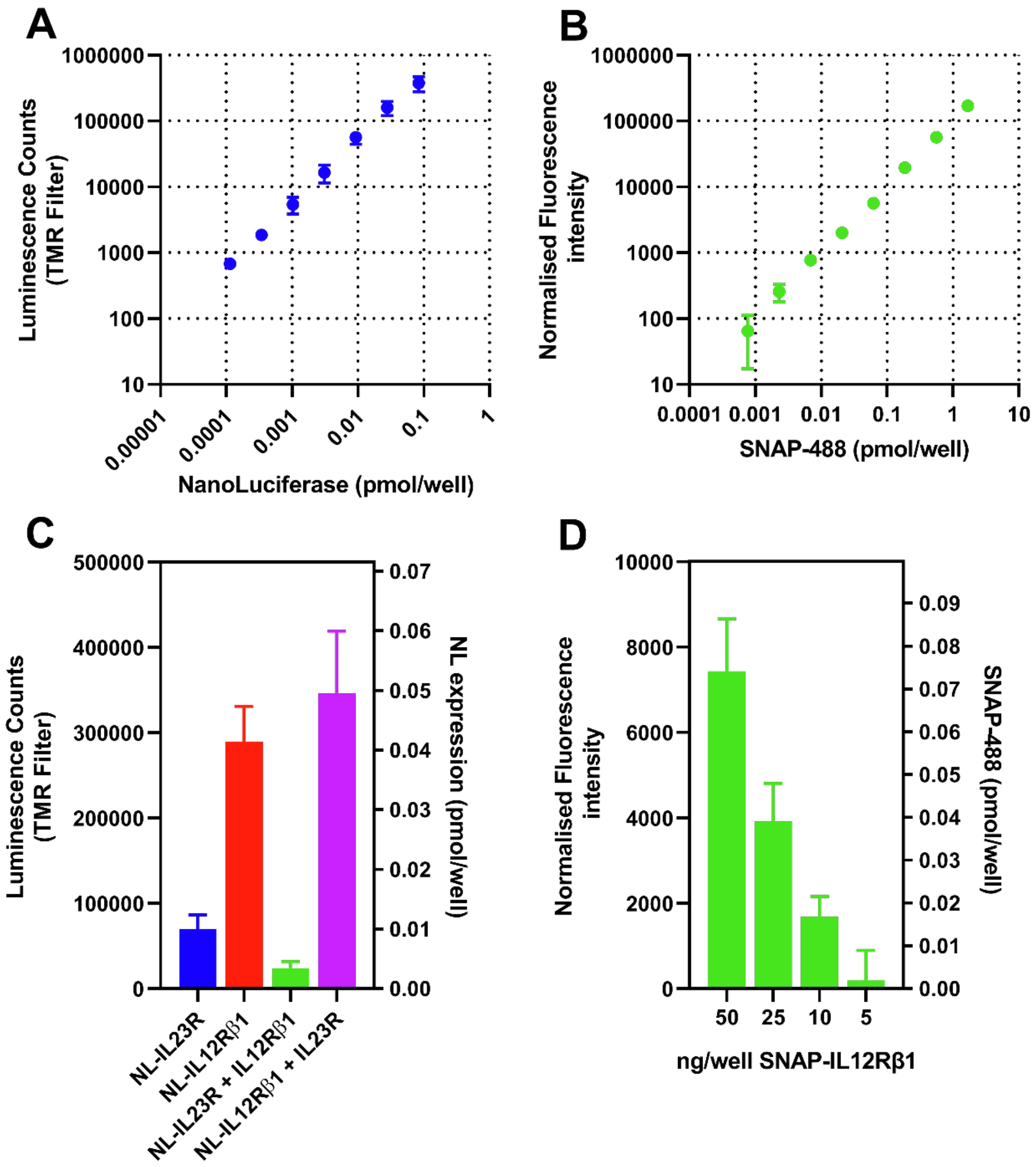

Figure S4 (Related to Figure 5 and Figure 7): Standard curves of purified NL and SNAP-AF488 allows the prediction of experimental construct concentration. (A) A standard curve of purified NL. Mean values with SEM from 3 independent experiments. (B) A standard curve of SNAP-AF488, normalised to background fluorescence intensity. Mean values with SEM from 4 independent experiments. (C) Transformation of luminescence values from HEK293T cells expressing equal quantities of NL-linked constructs to predicted NL expression values using the curve shown in (A). Transformation of fluorescent intensity values of cells transfected with varying concentrations of SNAP-IL12Rβ1 shown in Figure 7 to estimated SNAP-488 expression levels using the curve shown in (B).

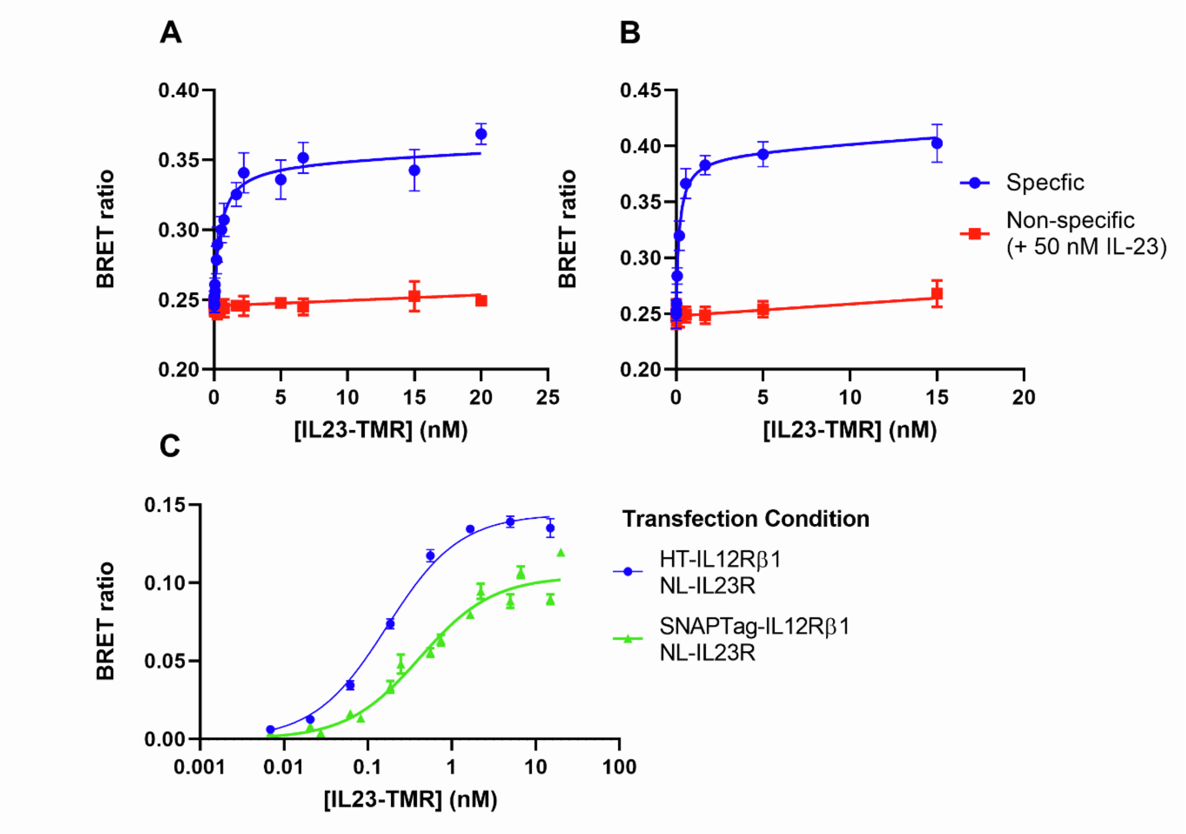

Figure S5 (Related to Figure 7): Fusion of SNAPTag or HaloTag to IL12Rβ1 does not block ligand binding. (A-B) The binding of IL23-TMR to cells expressing NL-IL23R and either HaloTag (A) or SNAPTag (B) fused IL12Rβ1, in the presence or absence of 50 nM unlabelled IL-23. Data is mean with SEM from five (A) or three (B) independent experiments. (C) The specific binding data shown in (A) and (B) with non-specific subtracted.

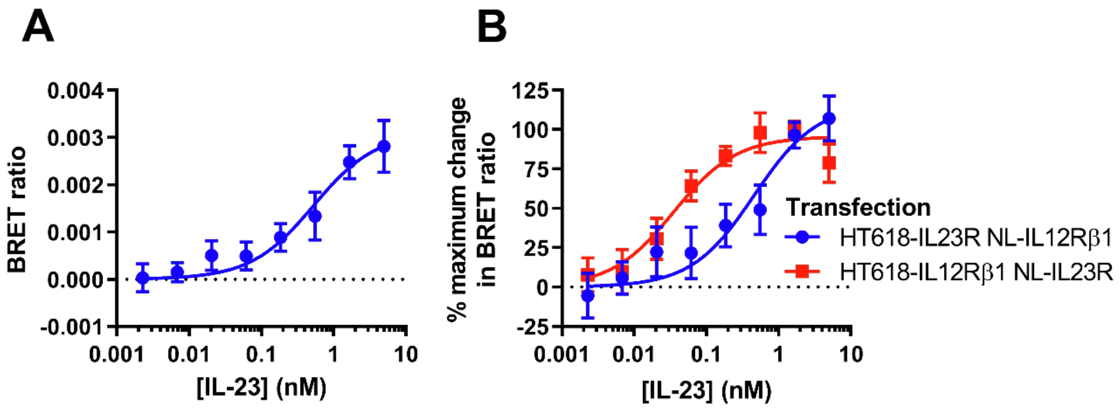

Figure S6 (Related to Figure 7): IL-23 mediated change in intra-receptor BRET measured using NL-IL12Rβ1 and HT618-IL23R. (A) The change in intra-receptor BRET between NL-IL12Rβ1 and HT618-IL23R expressed on HEK293T cells after incubation with unlabelled IL-23. Background normalised mean ± SEM BRET ratio values obtained from 4 independent experiments. (B) The data shown in (A) normalised to % change in BRET with equivalent data from cells transfected with HT618-IL12Rβ1 and NL-IL23R for reference.
