# Supplementary material for: Probing the binding of interleukin-23 to individual receptor components and the IL-23 heteromeric receptor complex in living cells using NanoBRET
Source: Cell Chem Biol. 2022 Jan 20;29(1):19–29.e6. doi: 10.1016/j.chembiol.2021.05.002 (PMC8790524; doi:10.1016/j.chembiol.2021.05.002)
Supplement: Document S2. Article plus supplemental information [file mmc2.pdf]

Article

Cell Chemical Biology

Probing the binding of interleukin-23 to individual receptor components and the IL-23 heteromeric receptor complex in living cells using NanoBRET

Graphical abstract

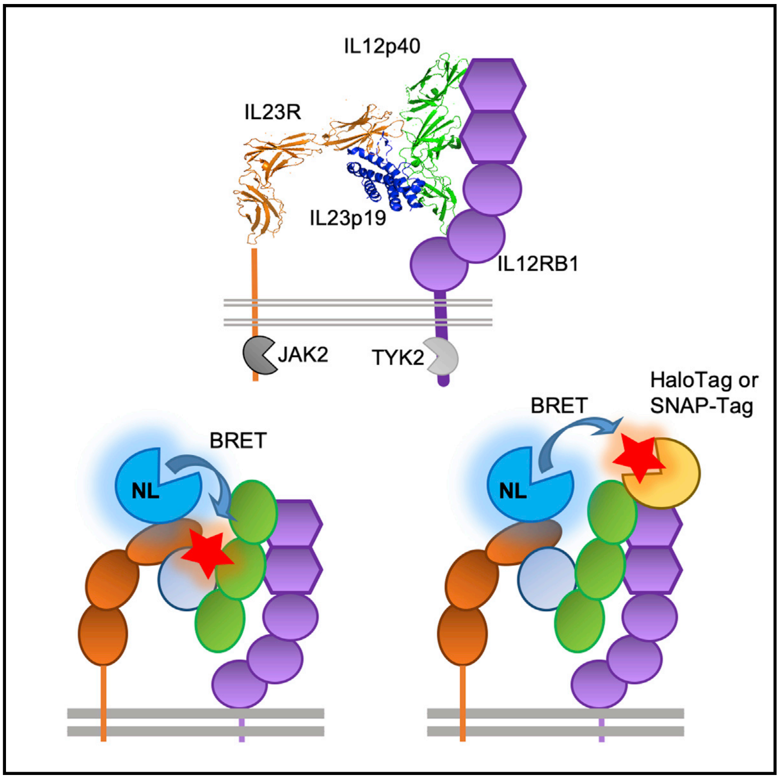

Authors

Charles S. Lay, Angela Bridges, Joelle Goulding, Stephen J. Briddon, Zoja Soloviev, Peter D. Craggs, Stephen J. Hill

Correspondence

Peter.d.craggs@gsk.com (P.D.C.),
Stephen.hill@nottingham.ac.uk (S.J.H.)

In brief

Lay et al. measure the binding of TAMRA-labeled IL-23 to NanoLuciferase fused receptor subunits, revealing a high-affinity binding site formed from a heteromer of IL23R and IL12Rβ1. NanoBRET was also used to measure the interaction of subunits, demonstrating that IL-23 binding causes a conformational change of pre-formed receptor heteromers.

Highlights

- NanoBRET assays were created to monitor the interaction of IL-23 with its receptor
- The affinities of IL-23 for IL23R, IL12Rβ1 and heteromer complexes were measured
- Receptor monomers were found to associate in complexes in the absence of IL-23
- IL-23 binding induced a rearrangement of the N-terminal domains of the receptor

Lay et al., 2022, Cell Chemical Biology *29*, 19–29
January 20, 2022 © 2021 The Author(s). Published by Elsevier Ltd.
https://doi.org/10.1016/j.chembiol.2021.05.002

CellPress

Cell Chemical Biology

CellPress
OPEN ACCESS

Article

Probing the binding of interleukin-23 to individual receptor components and the IL-23 heteromeric receptor complex in living cells using NanoBRET

Charles S. Lay,[1,2,3] Angela Bridges,[4] Joelle Goulding,[1,2] Stephen J. Briddon,[1,2] Zoja Soloviev,[4] Peter D. Craggs,[3,5,*] and Stephen J. Hill[1,2,6,*]

[1]Division of Physiology, Pharmacology and Neuroscience, School of Life Sciences, University of Nottingham, Nottingham NG7 2UH, UK
[2]Centre of Membrane Proteins and Receptors, Universities of Birmingham and Nottingham, The Midlands, UK
[3]Medicine Design, Medicinal Science and Technology, GlaxoSmithKline, Stevenage SG1 2NY, UK
[4]Protein and Cellular Sciences, Medicinal Science and Technology, GlaxoSmithKline, Stevenage SG1 2NY, UK
[5]GSK-Francis Crick Institute Linklabs, Medicinal Science and Technology, GlaxoSmithKline, Stevenage SG1 2NY, UK
[6]Lead contact
*Correspondence: Peter.d.craggs@gsk.com (P.D.C.), Stephen.hill@nottingham.ac.uk (S.J.H.)
https://doi.org/10.1016/j.chembiol.2021.05.002

SUMMARY

Interleukin-23 (IL-23) is a pro-inflammatory cytokine involved in the host defense against pathogens but is also implicated in the development of several autoimmune disorders. The IL-23 receptor has become a key target for drug discovery, but the exact mechanism of the receptor ligand interaction remains poorly understood. In this study the affinities of IL-23 for its individual receptor components (IL23R and IL12Rβ1) and the heteromeric complex formed between them have been measured in living cells using NanoLuciferase-tagged full-length proteins. Here, we demonstrate that TAMRA-tagged IL-23 has a greater than 7-fold higher affinity for IL12Rβ1 than IL23R. However, in the presence of both receptor subunits, IL-23 affinity is increased more than three orders of magnitude to 27 pM. Furthermore, we show that IL-23 induces a potent change in the position of the N-terminal domains of the two receptor subunits, consistent with a conformational change in the heteromeric receptor structure.

INTRODUCTION

Interleukin-23 (IL-23) is an important pro-inflammatory cytokine, produced by antigen-presenting cells, such as dendritic cells and macrophages, that plays a crucial role in host defense against bacterial and fungal pathogens (Verreck et al., 2004; Werner et al., 2011). IL-23 acts as a pre-requisite for the activation of T helper 17 (Th17) cells and has additional pro-inflammatory effects on natural killer T, innate lymphoid, and γδT cell types (Gaffen et al., 2014).

IL-23 is a disulfide-linked heterodimeric cytokine, an arrangement unique to the IL-12 family, of which there are three other reported members in man, IL-12, IL-27, and IL-35, with an additional member IL-39 identified in mice (Tait Wojno et al., 2019; Wang et al., 2012). The heterodimer is formed of a single domain four-helical bundle α subunit and a larger triplet domain β subunit composed of two type III fibronectin domains and a single immunoglobulin-like domain (Hasegawa et al., 2016). The α and β subunits of the IL-23 heterodimer are IL23p19 and IL12p40, respectively (Oppmann et al., 2000). The corresponding receptors for the IL-12 cytokines are heteromers formed from different combinations of five single transmembrane domain proteins. Each IL-12 heteromeric receptor component is thought to have affinity for a specific α or β subunit of each heterodimeric IL-12 family cytokine (Hasegawa et al., 2016). The specific receptor for the IL-23 cytokine is formed of the IL-12 receptor subunit β1 (IL12Rβ1) and the IL-23 receptor (IL23R), which are both needed for subsequent Janus kinase (JAK) activation and signaling (Parham et al., 2002). The formation of heteromers by IL-12 family cytokine receptors facilitates promiscuous pairing within the IL-12 family, leading to a diverse range of functional effects (Floss et al., 2020; Hasegawa et al., 2016). The archetypal example of this promiscuity is the dual use of the IL12p40 cytokine subunit and the IL12Rβ1 receptor subunit in both the IL-23 and IL-12 cytokine receptor complexes (Parham et al., 2002). Despite shared constituents, IL-23 and IL-12 mediate distinct pathways, with IL-23 inducing the expansion and maintenance of Th17 cells and IL-12, leading to differentiation of Th1 cells (Vignali and Kuchroo, 2012).

Both IL-12 and IL-23 became a major focus for therapeutic intervention when elevated expression of IL12p40 was observed in autoimmune disorders (Fassbender et al., 1998; Shigehara et al., 2003). This led to the first therapeutic to be developed for intervention in the IL-12 and IL-23 signaling pathways to be an anti-IL12p40 antibody (ustekinumab) (Benson et al., 2011). Further research utilizing genome-wide association studies

© 2021 The Author(s). Published by Elsevier Ltd.
This is an open access article under the CC BY license (http://creativecommons.org/licenses/by/4.0/).

demonstrated links between single-nucleotide polymorphisms in IL23p19 and IL23R and the incidence of ankylosing spondylitis, Crohn's disease (CD), ulcerative colitis (UC), and psoriasis among other disorders, identifying that IL-23 rather than IL-12 was involved in these diseases (Cargill et al., 2007; Dong et al., 2013; Duerr et al., 2006). Further evidence from murine studies demonstrated the IL-23 signaling pathway's role in autoinflammation (Cua et al., 2003; Langrish et al., 2005). Subsequently, anti-IL23p19 inhibitors have been developed, and antibody therapies are now licensed for the treatment of CD, UC, and psoriasis with ongoing trials for further autoinflammatory conditions (Chyuan and Lai, 2020). The importance of the IL-23 signaling pathway in other diseases has now been reported, including for cancer (Ngiow et al., 2013) and cardiovascular disease (Ye et al., 2020). Ustekinumab and other anti-IL23p19 inhibitors have been highly effective for the treatment of autoimmune disease; however, these treatments are not without issues, which include the reliance on administration by subcutaneous injection and immunogenicity (Jullien et al., 2015). These problems have led to the development of a class of small orally bioavailable peptide inhibitors of the IL-23 receptor (Kong et al., 2020) such as PTG-200, a competitive inhibitor of IL23R (Bourne, et al., 2016; Cheng et al., 2019).

Despite the intense therapeutic focus on IL-23 and the wider IL-12 family, there is a paucity of information regarding the specific mechanisms of the ligand-receptor interactions. The exact regions of IL-23 that interact with its receptor have only been defined relatively recently. Contrary to previous predictions based on the assembly of the closely related IL-6 receptor complex, the individual IL-23 receptor subunits appear to bind to distinct α and β chains of IL-23 (Schroder et al., 2015). Two recent studies have used X-ray crystallography to solve the structures of the IL-23:IL23R and IL-23:IL23R:IL12Rβ1(D1) complexes, respectively, with the latter group also determining cryogenic electron microscopy structures for the extracellular domains of IL-12 and IL-23 cytokine:receptor complexes (Bloch et al., 2018; Glassman et al., 2021). This work and further mutational studies demonstrated that IL-12 engages its receptor in a similar way to IL-23, with IL12p40 binding IL12Rβ1 and IL12p35 engaging IL12Rβ2 (Esch et al., 2020; Glassman et al., 2021). As part of the initial structural characterization of the IL23:IL23R complex, the affinity of IL-23 was measured for the purified extracellular domains of the IL-23 receptor using isothermal titration calorimetry (ITC). It was found that IL-23 has a higher affinity for the truncated extracellular domain of IL23R than the equivalent extracellular domain of IL12Rβ1, leading to the suggestion that the IL-23 receptor complex assembles via ligand-induced dimerization, with IL-23 first binding IL23R followed by the recruitment of IL12Rβ1 by the IL23R:IL-23 complex (Bloch et al., 2018).

To investigate the proposed model of IL-23 receptor complex formation, we have used NanoLuciferase (NL) bioluminescence resonance energy transfer (NanoBRET) (Machleidt et al., 2015; Stoddart et al., 2015) to measure the binding of a tetramethylrhodamine (TAMRA)-labeled variant of IL-23 to individual full-length IL-23 receptor subunits and heteromeric receptor complexes in living cells. We have also measured the level of constitutive association of receptor units and agonist-induced changes in receptor subunit conformation utilizing dual-tagged receptor variants that enable the measurement of intra-receptor NanoBRET.

RESULTS

Characterization of IL23-TMR and IL-23 receptor subunit fusion constructs

Preparation of fluorescently labeled IL-23 was carried out via incubation of purified recombinant IL-23 with NHS-ester-linked TAMRA. Following removal of the unreacted labeling reagent using a desalting column, the TAMRA-conjugated IL-23 (hereafter referred to as IL23-TMR) and the unlabeled IL-23 were assessed using liquid chromatography coupled with mass spectrometry (LC-MS; Figures S1 and S2). This analysis revealed that both the labeled and unlabeled cytokine samples were heterodimeric and uncontaminated with IL12p40 or IL23p19 monomers (Figure S2B). Furthermore, upon the application of dithiothreitol (DTT), the protein could be split into its constituent monomers by reduction of the linking disulfide bond (Figures S1 and S2). While the IL23p19 subunit closely matched its predicted molecular weight, the IL12p40 subunit gave several peaks differing by 162 Da, all with masses higher than that predicted from the protein's amino acid sequence (Table S1). This most likely corresponded to varying levels of post-translational modification (PTM) by N-linked glycosylation, a modification that has been previously reported for the IL12p40 subunit (Bohnacker et al., 2020). The unlabeled IL-23 heterodimeric complex exhibited the previously described heterogeneous pattern, with the most abundant peak corresponding to a 56,050-Da species (Figure S2).

In the IL23-TMR sample the most abundant species remained the unlabeled IL-23 at 56,050 Da; however, a single TAMRA-labeled 56,461-Da species and a double-labeled 56,876-Da species were also present. Trace amounts of three and four labeled species were also apparent from low-abundance peaks at 57,287 and 57,702 Da, respectively (Figure S2). Reduction of the IL23-TMR sample with DTT revealed that the IL23p19 subunit was labeled to a greater extent than the IL12p40 subunit (Figure S2A), with peak intensity analysis demonstrating that 64.9% of TAMRA labels were located on IL23p19. The primary constituents of the heterodimeric IL23-TMR fraction were determined by the relative abundance of the most intense PTM variant to be 52.3% unlabeled, 29.5% single labeled, and 18.2% double labeled.

To gain a further quantitative measure of the constituents of the labeled IL-23 mixture, we used fluorescence correlation spectroscopy (FCS) (Briddon et al., 2004) essentially as described by Kilpatrick et al. (2017). To measure the concentration of TAMRA-labeled particles in solution, we collected FCS fluctuations from freely diffusing IL23-TMR in a confocal measurement volume (Figure 1A). Subsequent autocorrelation analysis (Figure 1B), allowed calculation of the concentration of fluorescent particles (Briddon et al., 2004; Kilpatrick et al., 2017). Measured IL23-TMR concentrations were substantially lower than predicted, but the addition of 1 mg/mL BSA, which prevents non-specific interactions of the protein with the plate (Kilpatrick et al., 2017), increased the observed concentration (Figure 1D). Consequently, 1 mg/mL BSA was included in all subsequent IL-23 and IL23-TMR experiments. FCS

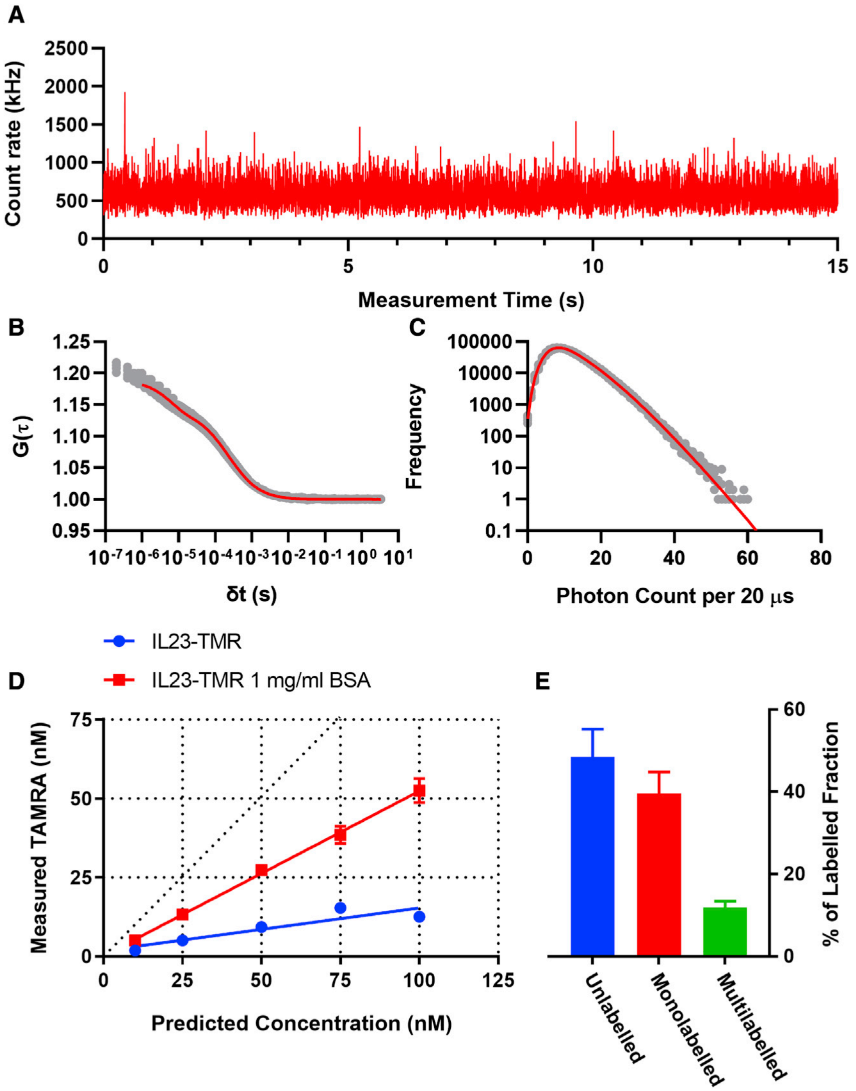

Figure 1. Characterization of labeled IL23-TMR using fluorescence correlation spectroscopy and photon-counting histogram analysis

(A) Example of the fluorescence fluctuations obtained over time for 100 nM IL23-TMR in the presence of 1 mg/mL BSA.

(B) Autocorrelation curves for four concurrently collected replicate fluorescence correlation spectroscopy (FCS) readings of 100 nM IL23-TMR in the presence of 1 mg/mL BSA, with a red line denoting the one-component free 3D fit of the data.

(C) Photon-counting histogram (PCH) of the data presented in (B) fit with a two-component model (red line).

(D) Analysis of IL23-TMR showing the concentration of TAMRA-labeled molecules measured by FCS in (B) versus the total concentration of IL-23 (initially quantified by absorbance at 280 nm), in the presence (red) or absence (blue) of 1 mg/mL BSA. Diagonal dotted line represents x = y. Each data point represents mean value ± SEM of 20 measurements from five independent experiments.

(E) Proportion of labeled IL-23 species. Total concentration of IL-23 was quantified by UV absorbance at 280 nm, and total concentration of TMR-labeled IL-23 was determined using the autocorrelation analysis demonstrated in (B). The proportion of different populations of labeled species was quantified using the two-component PCH analysis demonstrated in (C). Data shown are mean ± SEM of values obtained in five independent experiments.

measurements of IL23-TMR in the presence of BSA displayed a single diffusing species with a diffusion coefficient of 37.9 ± 1.8 $\mu m^2/s$ while that for TAMRA alone was 280 $\mu m^2/s$. By comparing the measured concentration of this IL23-TMR fluorescent species with the total concentration of IL-23 measured in the sample via absorbance at 280 nm, it was demonstrated that 51.5% of the IL-23 in the sample was fluorescently labeled with TAMRA.

The ratio of mono- to multi-labeled IL23-TMR particles was determined by calculating the average molecular brightness of individual fluorescent particles using a two-component photon-counting histogram analysis from the same intensity fluctuations (Chen et al., 1999; Figure 1C). It was found that the IL23-TMR fraction was 77% composed of a 163,983 ± 2,537 cpm/s brightness fraction and 23% composed of a 589,772 ± 5,557 cpm/s brightness fraction. The brightness of unconjugated TAMRA under these conditions was determined to be 301,461 ± 10,764 cpm/s. As the LC-MS spectra showed that the majority of labeled IL-23 is bound to just one TAMRA molecule, this demonstrated that reaction of the fluorophore with IL-23 caused significant quenching of its brightness, with a mean value almost 2-fold lower than that of unbound TAMRA. The second brightness component likely represents a mixture of IL-23 species, bound to two or more TAMRA molecules with a mean brightness formed both from variable label number and differing degrees of fluorescence quenching. As a result these components are hereafter referred to as mono- and multi-labeled IL-23. The overall proportions of these components when compared with the previously established total labeled IL-23 concentration were determined to be 39.6% mono-labeled and 11.9% multi-labeled IL-23, with a remaining 48.5% of the mixture being made up of unlabeled IL-23 (Figure 1B).

Cell surface expression of NL fusions of IL23R and IL12Rβ1 in HEK293T cells was confirmed using luminescence imaging and anti-NL immunocytochemical imaging, which showed that NL constructs were localized to the plasma membrane (Figure 2). Cell surface expression of HT-IL12Rβ1 and SNAP-IL12Rβ1 was confirmed by imaging cells expressing these constructs, which had been labeled by incubation with AlexaFluor 488 (AF488)-fused HaloTag or SNAP-tag substrate (Figure S3).

To calibrate NL luminescence and AF488 fluorescent intensity values in order to estimate expression levels, we made up and measured a standard curve of both purified NL and SNAP-AF488 substrate according to the same protocol that would be used in further experiments (Figure S4).

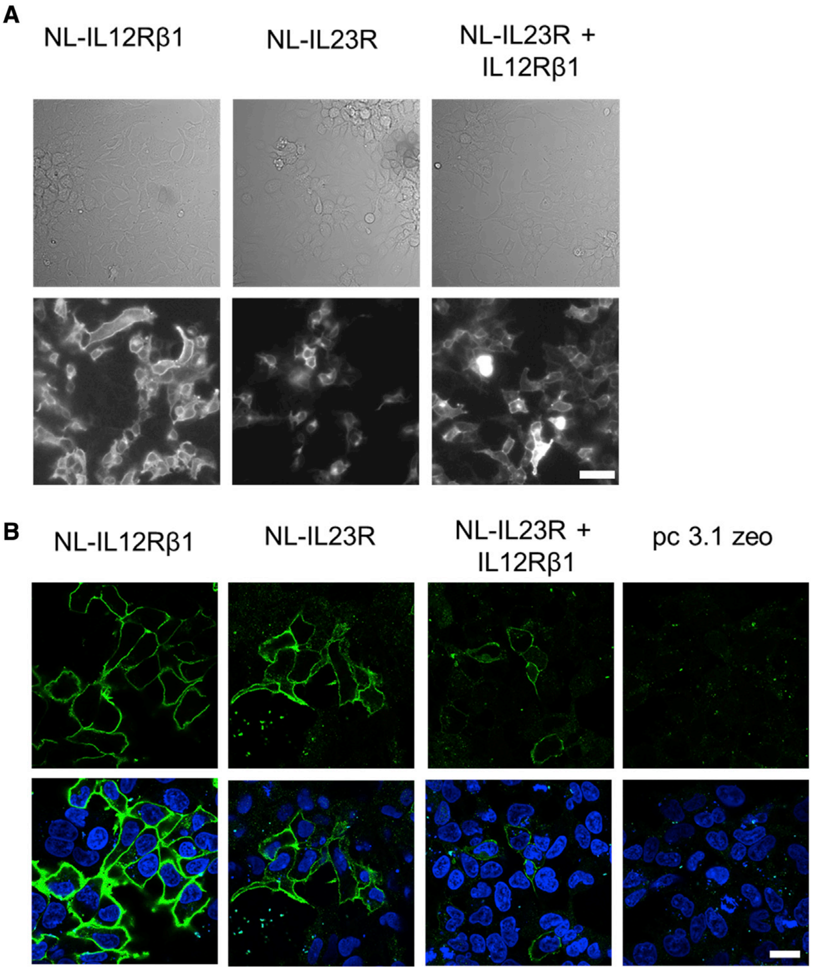

Figure 2. Expression of NanoLuciferase-tagged IL23R and IL12Rβ1 constructs

(A) Bright-field (top) and luminescence images (bottom) of HEK293T cells expressing NL-IL12Rβ1, NL-IL23R, and NL-IL23R co-expressed with IL12Rβ1 (collected with an Olympus LV200 Bioluminescence microscope). A 3-s exposure was used for NL-IL12Rβ1 transfected cells and a 10-s exposure for the other conditions. Images are representative of those collected in five independent experiments and were collected on the same experimental occasion. Scale bar represents 50 μm.

(B) Immunocytochemical imaging of transfected HEK293T cells labeled with an anti-NanoLuciferase antibody. Images are presented with (bottom) and without (top) Hoechst staining and are representative of those taken in three independent experiments. Images were taken on the same experimental day. Scale bar represents 20 μm.

To ascertain whether the fusion of NL to IL23R or the fusion of SNAP-tag or HaloTag to IL12Rβ1 had any effect on IL-23-induced signaling in HEK293T cells, we monitored phosphorylation of the downstream signal transducer and activator of transcription 3 (STAT3) transcription factor. Co-expression of the wild-type IL23R heteromer (IL23R and IL12Rβ1), followed by stimulation with varying concentrations of IL-23, led to determination of an EC_{50} = 269 ± 106 pM (n = 4) for cytokine stimulation of phosphorylated STAT3 (pSTAT3). This experiment was replicated utilizing co-transient transfections of either NL-IL23R and IL12Rβ1 or NL-IL23R and SNAP-tag fused IL12Rβ1 (SNAP-IL12Rβ1) or NL-IL23R and HaloTag fused IL12Rβ1, which led to the determination of potency values of 154 ± 46 pM (n = 4), 150 ± 43 pM (n = 4), and 199 ± 33 pM, respectively, for IL-23 stimulation of pSTAT3 (Figure 3). These results demonstrated that use of these fusion constructs did not significantly alter signal transduction induced by IL-23 ($p > 0.05$; one-way ANOVA).

Binding of IL23-TMR to IL12Rβ1, IL23R, and heteromers of both receptor subunits

The binding of IL23-TMR was measured using NanoBRET in HEK293T cells transiently expressing NL fusions of IL23R or IL12Rβ1 in the presence and absence of the corresponding untagged co-receptor. Increasing concentrations of IL23-TMR resulted in an increase in the BRET signal that was composed of saturable specific and linear non-specific binding components (Figure 4). Non-specific binding was determined in the presence of an excess of unlabeled IL-23; 1 μM was used for NL-IL23R and NL-IL12Rβ1 transfected cells and 50 nM was used for NL-IL23R co-expressed with IL12Rβ1. IL23-TMR had a greater affinity for NL- IL12Rβ1 (K_D = 30.1 ± 5.5 nM; n = 5) than NL-IL23R (K_D = 222.2 ± 71.1 nM; n = 5) when the constructs were expressed in isolation (Figures 4A and 4B). However, when NL-IL23R was co-expressed with unlabeled IL12Rβ1, the dissociation constant of IL23-TMR to the heteromeric receptor complex was markedly decreased to 27.0 ± 3.6 pM (n = 5), which was significantly different from the affinity of IL23-TMR to cells expressing NL fused monomers (unpaired t test; Figure 4C).

The dissociation constant obtained for IL23-TMR binding to NL-IL12Rβ1 in the presence of unlabeled IL23R was higher (46.7 ± 16.1 nM, n = 3; Figure 5B) and similar to the value obtained with NL-IL12Rβ1 alone. However, comparison of the luminescence intensities of cells expressing NL-IL12Rβ1 and NL-IL23R indicated that NL-IL12Rβ1 achieved a much higher expression level in transient transfections than that obtained with NL-IL23R (0.0518 ± 0.0066 versus 0.0125 ± 0.0027 pmol/well, respectively; Figure 5A). The higher dissociation constant obtained for NL-IL12Rβ1 in the presence of unlabeled IL23R is therefore likely to be a consequence of the NL-IL12Rβ1:IL23R complex only representing a small proportion of the total binding of IL23-TMR to NL-IL12Rβ1. To investigate this further, we undertook transfections using a lower (1:10) transfection ratio of NL-IL12Rβ1 and IL23R. The affinity of IL23-TMR under these conditions was 19-fold higher (K_D = 647 ± 79.5 pM, n = 5; Figure 5B) and consistent with a higher proportion of NL-IL12Rβ1:IL23R complexes present under these experimental conditions.

To ascertain whether the fusion of N-terminal tags to IL12Rβ1 blocked the binding of IL23-TMR, BRET binding experiments

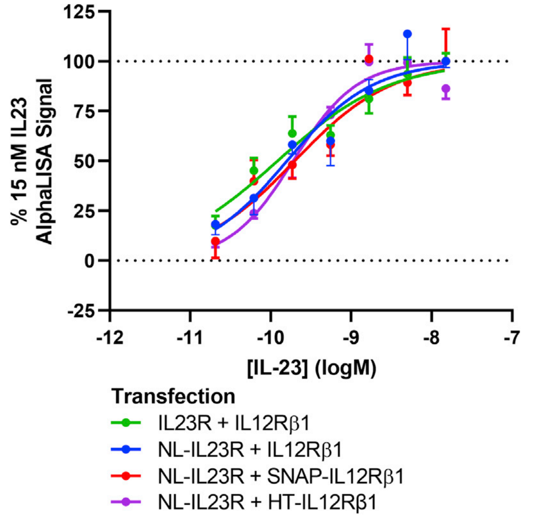

Figure 3. The effect of N-terminal NanoLuciferase, HaloTag, or SNAP-tag additions to IL-23 receptor subunits on IL-23-induced STAT3 phosphorylation

STAT3 phosphorylation induced in HEK293T cells transfected with different tagged variants of the IL-23 receptor after a 30-min incubation with increasing concentrations of IL-23. Data are expressed as a percentage of the response obtained with 15 nM IL-23. Values are mean ± SEM from four or three (NL-IL23R and HT-IL12Rβ1) independent experiments.

analogous to those previously detailed were carried out using cells expressing NL-IL23R and HT-IL12Rβ1 or SNAP-IL12Rβ1. IL23-TMR bound to cells expressing both receptor fusion constructs with an affinity of 179 ± 8 pM and 454 ± 43 pM to the HT-IL12Rβ1 and SNAP-IL12Rβ1 conditions, respectively (Figure S5).

Binding affinity of unlabeled IL-23 for the NL-IL23R:IL12Rβ1 receptor complex

To measure the affinity of unlabeled IL-23 for the IL-23 receptor complex, we undertook NanoBRET competition experiments using NL-IL23R and IL12Rβ1 co-transfected into HEK293T cells. This assay was carried out using several concentrations of IL23-TMR and increasing concentration of unlabeled IL-23, whereby the IC_{50} determined for unlabeled IL-23 was shifted to higher concentrations (with increasing concentrations of IL23-TMR) consistent with a competitive interaction (Figures 6A and 6B; Cheng and Prusoff, 1973). However, at the highest concentration of IL-23 used (3 nM) the decrease in potency reached a limiting value indicative of a more complex interaction (Figures 6A and 6B). Analysis of the data obtained with lower concentrations of IL23-TMR yielded a mean K_i for IL-23 of 31.6 ± 7.7 pM (n = 13), which was not significantly different from the pre-determined value for the K_D of IL23-TMR (unpaired t test). In addition, a $K_{i\ IL\text{-}23}$ of 27.2 pM and a $K_{D\ IL23\text{-}TMR}$ of 35.1 pM were obtained from a linear plot of the relationship between IL23-TMR concentration and the corresponding IC_{50} values of unlabeled IL-23 (Figure 6B).

IL-23-induced changes in the position of the N termini of IL12Rβ1 and IL23R

To ascertain the level of constitutive association between IL12Rβ1 and IL23R, we transfected HEK293T cells with varying concentrations of N-terminal fused SNAP-IL12Rβ1 plasmid with a constant concentration of NL-IL23R plasmid. The BRET signal between NL and AF488 bound to the SNAP-tag was then measured in the presence and absence of 5 nM IL-23. BRET was observed between the subunits both with and without IL-23, with the signal amplitude in the absence of IL-23 being 54.5% ± 3.9% that of the signal with IL-23. The BRET signal was saturable with increasing SNAP-IL12Rβ1 plasmid concentration (Figure 7A); however, the level of SNAP-IL12Rβ1 expression increased in a linear fashion with plasmid concentration (Figure 7B), as quantified by fluorescent intensity measurements before substrate addition. This demonstrated that the increase in BRET signal was due to specific interactions of the constructs rather than non-specific BRET in the membrane. The BRET ratio obtained for the interaction between NL-IL23R and SNAP-IL12Rβ1 was significantly increased by 5 nM IL-23 ($p < 0.0001$; t test). The ng of SNAP-IL12Rβ1 cDNA transfected per well could be further transformed into the pmol/well of SNAP-IL12Rβ1 construct through the use of the SNAP-tag AF488 curve outlined in Figure S4. This transformation (Figures 7C and 7D) enabled the calculation of the $BRET_{50}$ values of 0.0167 ± 0.0094 and 0.0840 ± 0.0409 pmol/well SNAP-IL12Rβ1 per well in the presence and absence of 5 nM IL-23, respectively.

To determine how the receptor subunit proximity or orientation changed during the formation of the ligand-bound IL-23 receptor complex, we carried out a BRET assay whereby HEK293T cells were co-transfected with equal concentrations of NL-IL23R and N-terminal SNAP-tag or HaloTag fused IL12Rβ1. The receptor was fluorescently labeled by addition of SNAP-tag AF488 substrate to the SNAP-tag or HaloTag 618 (HT618) ligand to the HaloTag. In both the SNAP-tag and HaloTag-IL12Rβ1 conditions there was a clear change in BRET ratio after incubation with increasing concentrations of unlabeled IL-23 (Figure 7D). The EC_{50} values of IL-23 for these responses were 42.9 ± 10.3 pM (n = 5) and 43.6 ± 15.8 pM (n = 5) for the SNAP-tag and HaloTag assays, respectively (no statistical difference; unpaired t test; Figure 7D). However, while the SNAP-IL12Rβ1 assay gave an increase in BRET signal following addition of IL-23, in the HaloTag-IL12Rβ1 assay the BRET signal decreased with increasing concentrations of IL-23 (Figure 7E). A comparable BRET assay was also established using HEK293T cells transfected with both NL-IL12Rβ1 and HaloTag-IL23R. This assay demonstrated an increase in BRET with increasing concentrations of IL-23 but gave a higher EC_{50} value of 457 ± 209 pM (Figure S6). Taken together, these data are consistent with a change in conformation of the IL23-R:IL12Rβ1 complex rather than an increase in the formation of these complexes following agonist addition.

DISCUSSION

The IL-23 cytokine and its receptor are important therapeutic targets (Verreck et al., 2004; Werner et al., 2011). However, many aspects relating to the mechanisms by which the IL-23:IL23R:IL12Rβ1 protein complex is formed remain to be fully elucidated. In the present study, NanoBRET was utilized to measure the affinity of fluorescently labeled IL-23 to NL fusions of the full-length individual IL-23 and IL12Rβ1 receptor subunits and the full-length IL-23 receptor heteromeric complex in living cells.

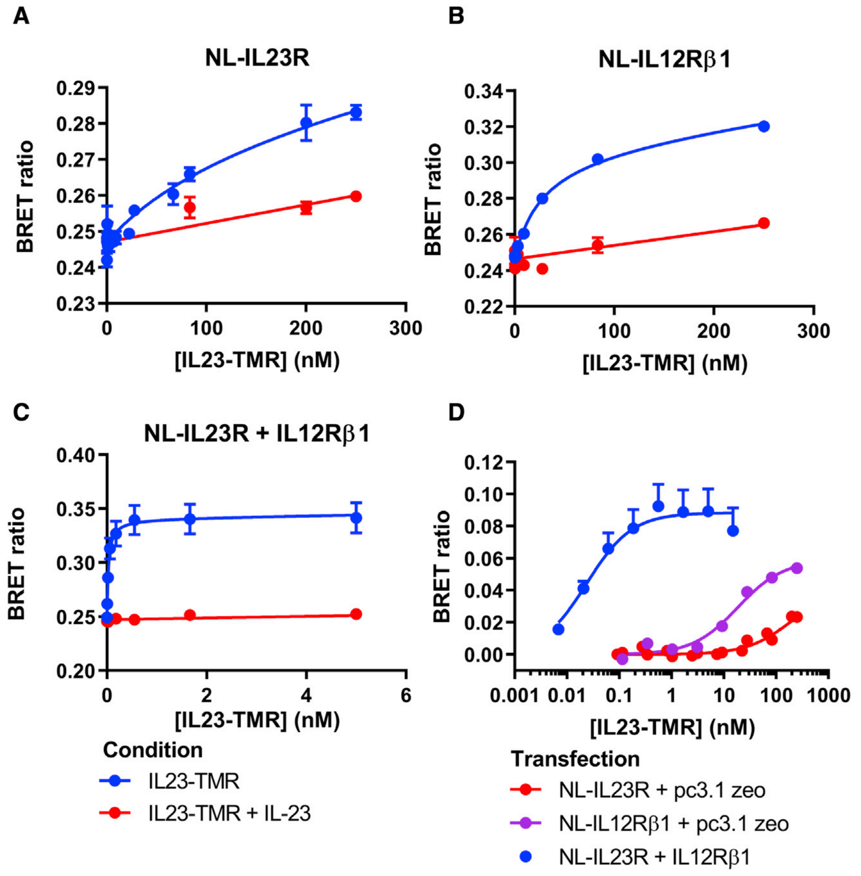

Figure 4. Binding of IL23-TMR to NL-IL12Rβ1, NL-IL23R, and NL-IL23R co-expressed with untagged IL12Rβ1

(A–C) Binding of increasing concentrations of IL23-TMR to HEK293T cells expressing either (A) NL-IL23R, (B) NL-IL12Rβ1, or (C) NL-IL23R and IL12Rβ1 in the presence (red) or absence (blue) of an excess of unlabeled IL-23. The concentration of unlabeled IL-23 used was 1 μM (A and B) and 50 nM (C). Data points are mean raw BRET ratio values ± SEM from five independent experiments.

(D) The combined specific binding data from (A) to (C) after subtraction of non-specific binding.

A previous study that characterized the interaction of IL-23 with the purified extracellular domains of the IL23R and IL12Rβ1 receptor subunits using ITC indicated that IL-23 has a much higher affinity for the extracellular domain of IL23R (44 nM) than the extracellular domain of IL12Rβ1 (2 μM) (Bloch et al., 2018). These observations led Bloch and colleagues to suggest that the binding of IL-23 involved a sequential mechanism whereby IL-23 binds first to the IL23R subunit and then induces a dimerization step between the IL23R:IL-23 complex and IL12Rβ1, leading to the final complex (Bloch et al., 2018).

In the present study, we determined that the dissociation constant of IL23-TMR binding to full-length cell surface-expressed NL-IL23R was 222.2 ± 71.1 nM. However, the dissociation constant of IL23-TMR binding to the full-length NL-IL12Rβ1 in intact cells was 30.1 ± 5.5 nM. This is nearly an order of magnitude lower than the value obtained for IL23R and suggests that the sequence of events identified by Bloch et al. (2018) may need to be re-evaluated. The higher affinity of IL23-TMR for IL12Rβ1 in live cells is also consistent with previous reports that cells expressing IL12Rβ1 have a 2- to 5-nM affinity for the IL-12 cytokine (Chua et al., 1994). This observation is pertinent to the current study, given that IL-12 and IL-23 heteromers share the IL12p40 cytokine subunit and have both been shown to engage their receptors in a similar manner (Glassman et al., 2021).

When NL-IL23R was co-expressed in cells alongside unlabeled IL12Rβ1, the affinity for IL23-TMR was increased by nearly four orders of magnitude to 27.0 ± 3.6 pM. This suggests that a third, high-affinity binding site for IL23-TMR is formed by oligomeric complexes containing both IL23R and IL12Rβ1 on the cell surface. To further characterize this third binding site, we measured the affinity of unlabeled IL-23 in cells expressing both NL-IL23R and IL12Rβ1 using a competition binding assay. The results of these competition experiments confirmed that the IL-23:IL12Rβ1 complex had a high affinity for the untagged IL-23 cytokine, K_D = 27.2 ± 12.0 pM, which was similar to that measured for IL23-TMR. In the context of a growing list of reported IL23R antagonists (Cheng et al., 2019; Kong et al., 2020; Kuchař et al., 2014; Pandya et al., 2020; Quiniou et al., 2014), this assay should be a useful tool for future screening efforts to identify and develop IL-23 receptor inhibitors.

To determine whether IL23R and IL12Rβ1 proteins can form constitutive heterodimers in the absence of ligand in HEK293T cells, and to what extent this is influenced by agonist treatment, we compared BRET measures between NL-tagged IL23R and a fluorescently labeled SNAP-tag fusion of IL12Rβ1. These experiments demonstrated that IL12Rβ1 and IL23R can form dimers in the absence of IL-23, as demonstrated by the saturable increase in BRET ratio obtained with increasing amounts of transfected SNAP-IL12Rβ1. Furthermore, following the addition of IL-23 (5 nM) there was an increase in the BRET ratio obtained for the interaction between NL-IL23R and SNAP-IL12Rβ1. Addition of IL-23 produced a concentration-dependent increase in BRET ratio in HEK293T cells transfected with a 1:1 ratio of NL-IL23R and SNAP-IL12Rβ1. This interaction had an EC_{50} value of 42.9 pM that corresponded well with the high-affinity binding site measured in IL-23 competition assays (K_D = 31.6 pM) when both NL-IL23R and IL12Rβ1 were expressed together in the same cells. However, while in the SNAP-IL12Rβ1 assay the BRET signal increased with IL-23 binding, in the HaloTag-IL12Rβ1 assay the BRET signal decreased following agonist binding. The IC_{50} value for this inhibitory effect of IL-23 was very similar (43.6 pM) to the EC_{50} value obtained for the increase in BRET signal in the SNAP-IL12Rβ1 assay. Furthermore, when the HaloTag was placed on IL23R and the NL tag was added to IL12Rβ1, this

CellPress
OPEN ACCESS

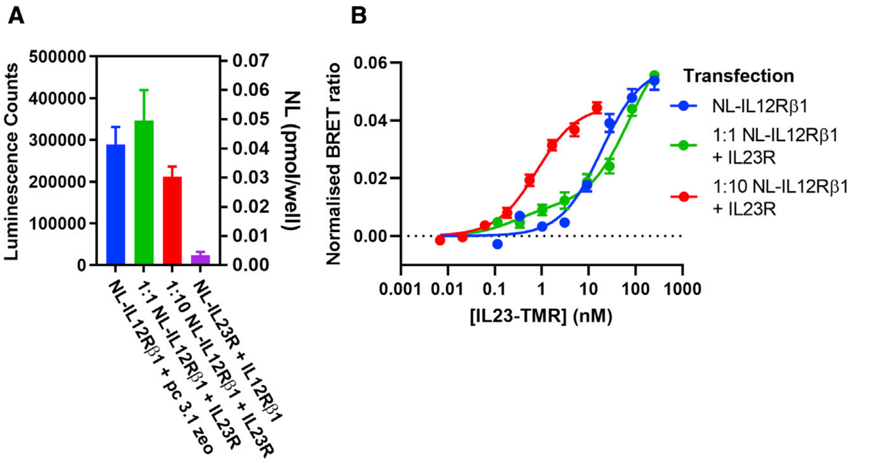

Figure 5. Impact of expression level on ligand-binding characteristics of NL-IL12Rβ1

(A) Luminescence signal measured from HEK293T cells following transient transfection. Corresponding NL (pmol/well) levels are calculated from a purified NL standard curve (Figure S4). Values are expressed as mean ± SEM from five or three (1:1 NL-IL12Rβ1 + IL23R) independent experiments.

(B) Binding of IL23-TMR to combinations of NL-IL12Rβ1 and IL23R at different transfection ratios in HEK293T cells. Values show mean ± SEM cells of the BRET ratios obtained after subtraction of non-specific binding. The concentration of IL-23 used to define non-specific binding was 1 μM (blue and green) and 100 nM (red). Data were obtained from three (green) or five (red and blue) separate experiments. The data for 1:1 NL-IL12Rβ1 + IL23R (green circles) were fitted to a two-site binding curve, which yielded K_D values of 28 pM and 72 nM with the high-affinity site accounting for 16.2% of the maximum specific binding. For these data, the two-site binding curve was a significantly better fit than to a single-site saturation curve ($p < 0.0001$; partial F test).

IL-23-induced decrease in BRET was changed to a large increase in the BRET signal.

The resonance energy transfer that occurs between two labeled proteins depends very much on both proximity (<10 nm) and orientation of the acceptor and donor species (Dacres et al., 2012). In the case of receptors that are labeled with both a donor and acceptor species, this can lead to agonist-induced increases or decreases in BRET as a consequence of conformational changes induced by agonist binding. This has been demonstrated particularly well with intra-molecular FRET sensors for G-protein-coupled receptors (Perpiñá-Viciano et al., 2020). Taken together, the data obtained for IL-23:IL12Rβ1 oligomerization indicates that binding of IL-23 to a high-affinity binding site formed by the oligomeric complex triggers a conformational change in the N-terminal domains of the IL-23 receptor components. This is the simplest explanation for the positive and negative BRET signals obtained following IL-23 addition for the interaction between IL23R and IL12Rβ1 with different tag arrangements. The SNAP-tag is a 19.4-kDa protein and the HaloTag is a 33-kDa protein (Crivat and Taraska, 2012), so it is very likely that their orientation with respect to the 19-kDa NL on NLuc-tagged proteins can change in different ways as a result of conformational changes induced by IL-23.

We observed a decrease in the potency of IL-23-induced conformational change when using cells transfected with the HT-IL23R and NL-IL12Rβ1 tagging conformation; however, for cells transfected with the constructs NL-IL23R and SNAP-IL12Rβ1 we measured no change in the potency of IL-23-induced STAT phosphorylation compared with the untagged control. Notwithstanding any subtle effects of the NL fusions and fluorescent labeling on IL-23 binding affinity, it is clear that the NanoBRET technique has enabled the detection of binding events that are at a far higher order of affinity than any of those previously measured at the receptor using untagged but truncated purified protein (Bloch et al., 2018). Furthermore, we have shown that SNAP fusions of IL12Rβ1 and NL fusions of IL23R do not abrogate the ability of the receptor to initiate signal transduction through the phosphorylation of STAT3. The high-affinity binding site identified in this work is in agreement with the EC_{50} values we measured for downstream phosphorylation of STAT3 and others measured for the IL-23-induced phosphorylation of STAT5 (EC_{50} = 28 pM; Varghese et al., 2020).

A consensus mechanism of receptor activation for the wider IL-12 cytokine family of receptors has yet to be elucidated. Within the cytokine receptor family, initial suggestions of ligand-induced dimerization (Cochet et al., 1988) have been superseded in many instances by a mechanism involving

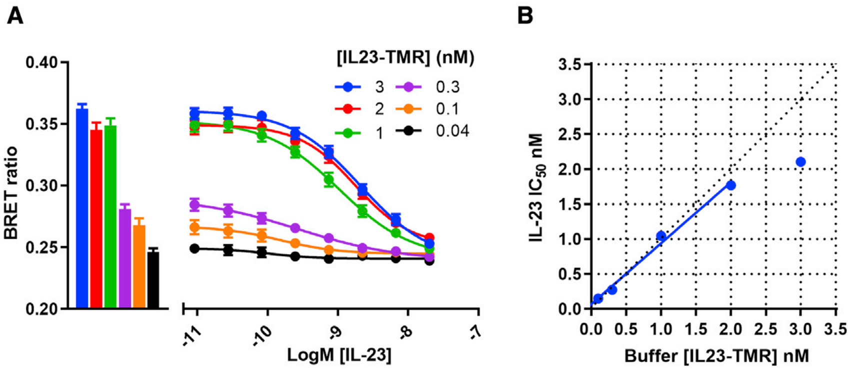

Figure 6. Determining the affinity of unlabeled IL-23 for the IL-23 receptor

(A) HEK293T cells expressing NL-IL23R and IL12Rβ1 were incubated with varying concentrations of IL23-TMR as shown in the bar chart (left) and increasing concentrations of unlabeled IL-23 (right). Data represent mean ± SEM of the raw BRET ratios obtained from five independent experiments.

(B) IL-23 IC_{50} values derived in (A) plotted against the buffer concentration of IL23-TMR, with all points except that for 3 nM IL23-TMR fitted by linear regression.

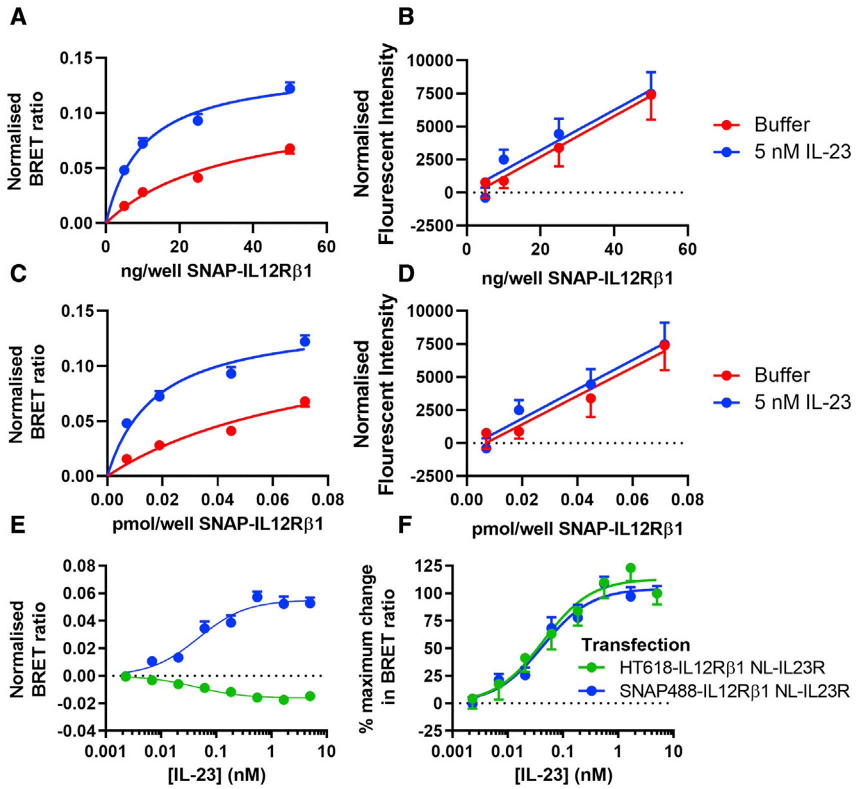

Figure 7. Association of IL12Rβ1 and IL23R in the absence of IL-23 with changes in the position of the N-terminal regions of IL12Rβ1 and IL23R following binding of IL-23

(A) Effect on the BRET signal of transfecting increasing concentrations of SNAP-IL12Rβ1 with 50 ng of NL-IL23R into cells. The SNAP-IL12Rβ1 was labeled with SNAP-tag-AF488 substrate and BRET between NL-IL23R and SNAP-IL12Rβ1 was then monitored in the presence or absence of 5 nM IL-23. Data are Mean ± SEM from five independent experiments. The BRET signal was normalized to cells expressing NL-IL23R in isolation.

(B) Fluorescence intensity signals from laser-excited SNAP-tag bound AF488 taken before the BRET readings in (A), normalized to the fluorescence intensity signal of cells expressing NL-IL23R in isolation.

(C and D) Data shown in (A) and (B), respectively with the x axis transformed to pmol/well SNAP-IL12Rβ1 as calculated from the normalized fluorescent intensity values using an SNAP-AF488 substrate standard curve (Figure S4).

(E) Intra-receptor BRET between HT618-IL12Rβ1 (green) or SNAP488-IL12Rβ1 (blue) co-expressed with NL-IL23R on HEK293T cells after incubation with increasing concentrations of unlabeled IL-23. Data show mean ± SEM of background-corrected BRET signals from five independent experiments.

(F) Data from (E) normalized to percentage of the maximum inhibition or induction of BRET.

conformational change and the presence of pre-formed inactive dimeric receptor complexes (Chua et al., 1994; Gent et al., 2002; Couturier and Jockers, 2003; Schuster et al., 2003). These receptors can then be activated by a rotation of the transmembrane domains (reviewed by Maruyama, 2015), which results in a rearrangement of pre-dimerized *trans*-inhibiting JAKs allowing phosphorylation and signaling (Waters et al., 2014). If this is the case for the IL-23 receptor, it is unlikely that the interaction occurs within the extracellular domains of the receptor subunits alone, as it has previously been demonstrated that purified IL23R and IL12Rβ1 extracellular domains have no affinity for each other (Bloch et al., 2018) and even that these domains can be replaced entirely by nanobodies and still initiate signaling with the addition of a synthetic dual antigenic ligand (Engelowski et al., 2018).

While the present study contradicts the previous report of the ligand-induced dimerization of the IL-23 receptor, there is evidence to support the existence of pre-dimerized receptor complexes at the IL-23 receptor, within the wider IL-12 family and at other closely related receptors. Sivenesan and colleagues used IL-23 receptor C-terminal fusions of split Renilla luciferase expressed in HEK293 cells to demonstrate luminescence in the absence of IL-23 and used this as evidence to argue that the IL-23 receptor is constitutively associated in the absence of ligand, similarly to the related erythropoietin receptor (Remy et al., 1999; Sivanesan et al., 2016).

The initial study outlining the discovery of the IL-12 receptor reported cytokine binding sites of three different affinities on live cells, and the authors proposed that the highest-affinity 5- to 20-pM site was formed by a pre-formed receptor dimer (Chua et al., 1994). In addition, the deletion of the extracellular stalk region of IL23R has been shown to cause the formation of IL23R receptor subunit complexes that signal in the absence of ligand (Hummel et al., 2017); this could be evidence for an inhibitory domain that ordinarily prevents signaling in constitutively formed dimers. The closely related IL-6 receptor, which includes a gp130 subunit highly homologous to IL12Rβ1 (Chua et al., 1994), has also been shown to undergo ligand-independent dimerization (Schuster et al., 2003).

This study has outlined the application of a proximity-based technique for the IL-12 cytokine family. This approach could be further developed to gain insights into the kinetics of cytokine binding and receptor assembly and be used to assess any differences in interactions caused by disease-relevant mutations. The successful application of this technology to the IL-23 receptor also highlights its utility to measure similar interactions at other related cytokine receptors that remain equally underinvestigated, with IL-12 being the only one of the four IL-12 family cytokines to have a reported cellular affinity (Chua et al., 1994).

The IL-23 cytokine and its receptor are a target of high therapeutic interest. However, the binding mechanism, an important piece of information for drug discovery, has not yet been defined on living cells. This study defines the affinity of IL-23 to its full-length receptor in living cells, demonstrating that the current ligand-induced dimerization hypothesis of IL-23 receptor assembly is unlikely and that a conformational change-based activation mechanism is the more probable situation. The

implications of this are that two further protein-protein interaction sites could exist for drug discovery, firstly the dimeric IL23R: IL12Rβ1 interface with IL-23 and secondly the IL23R interface with IL12Rβ1.

SIGNIFICANCE

The IL-23 receptor is an important therapeutic target for autoinflammatory conditions such as Crohn's disease and psoriasis; however, the mechanism of receptor activation has yet to be fully defined. Using NanoBRET we measured the interaction of IL23-TMR with NL fusions of the full-length IL-23 receptor subunits expressed in cells and report that IL23-TMR had a higher affinity for NL-IL12Rβ1 compared with that for NL-IL23R. In addition, we observed that co-expression of NL-IL23R with IL12Rβ1 created a binding site with an affinity far higher than those measured for NL-IL23R or NL-IL12Rβ1 alone. This heteromeric complex could be formed in the absence of added ligand, suggesting that this high-affinity binding site was constitutively present. Furthermore, the impact of IL-23 addition on the position of N-terminal regions of the constituent protomers was dependent on the nature of the tags used but was consistent with an IL-23-mediated change in receptor conformation. These results together suggest that IL-23 binds to pre-formed heteromers of both IL23R and IL12Rβ1, leading to a conformational change. This finding is significant, as it has been reported that the IL-23 receptor is activated by ligand-induced dimerization with initial binding to the IL23R subunit. The findings of this study have direct implications for drug discovery both by outlining a methodology through which to characterize IL-23 receptor antagonists and by revealing that the IL23R:IL12Rβ1 interface could be a good target to disrupt IL-23 receptor heteromer formation and signaling.

STAR★METHODS

Detailed methods are provided in the online version of this paper and include the following:

- KEY RESOURCES TABLE
- RESOURCE AVAILABILITY
  - Lead contact
  - Materials availability
  - Data and code availability
- EXPERIMENTAL MODEL AND SUBJECT DETAILS
- METHOD DETAILS
  - Materials
  - Molecular biology
  - Fluorescence correlation spectroscopy analysis of IL23-TMR
- QUANTIFICATION AND STATISTICAL ANALYSIS

SUPPLEMENTAL INFORMATION

Supplemental information can be found online at https://doi.org/10.1016/j.chembiol.2021.05.002.

ACKNOWLEDGMENTS

The work was funded by BBSRC (Industrial Case studentship to C.S.L., BB/S507027/1), Medical Research Council [grant number MR/N020081/1] and GSK.

AUTHOR CONTRIBUTIONS

S.J.H. and P.D.C. conceived the study and supervised the project. A.B. and C.S.L. generated reagents. S.J.H., P.D.C., S.J.B., J.G., Z.S., and C.S.L. participated in research design. C.S.L. conducted the experiments and performed the data analysis. All authors wrote or contributed to the writing of the manuscript.

DECLARATION OF INTERESTS

P.D.C, A.B., and Z.S. are employees of and shareholders in GSK.

Received: January 5, 2021
Revised: April 6, 2021
Accepted: May 4, 2021
Published: May 25, 2021

REFERENCES

Benson, J.M., Sachs, C.W., Treacy, G., Zhou, H., Pendley, C.E., Brodmerkel, C.M., Shankar, G., and Mascelli, M.A. (2011). Therapeutic targeting of the IL-12/23 pathways: generation and characterization of ustekinumab. Nat. Biotechnol. *29*, 615.

Bloch, Y., Bouchareychas, L., Merceron, R., Składanowska, K., Van den Bossche, L., Detry, S., Govindarajan, S., Elewaut, D., Haerynck, F., Dullaers, M., et al. (2018). Structural activation of pro-inflammatory human cytokine IL-23 by cognate IL-23 receptor enables recruitment of the shared receptor IL-12Rβ1. Immunity *48*, 45–58.

Bohnacker, S., Hildenbrand, K., Aschenbrenner, I., Müller, S.I., Bieren, J.E.von, and Feige, M.J. (2020). Influence of glycosylation on IL-12 family cytokine biogenesis and function. Mol. Immunol. *126*, 120–128.

Bourne, G.T., Bhandari, A., Cheng, X., Frederick, B.T., Zhang, J., Patel, D.V., and Lui, D. (2016). Oral Peptide Inhibitors of Interleukin-23 Receptor and Their Use to Treat Inflammatory Bowel Diseases, International Patent WO2016/011208, filed July 15, 2015 and granted April 18, 2017.

Briddon, S.J., Middleton, R.J., Cordeaux, Y., Flavin, F.M., Weinstein, J.A., George, M.W., Kellam, B., and Hill, S.J. (2004). Quantitative analysis of the formation and diffusion of A 1-adenosine receptor-antagonist complexes in single living cells. Proc. Natl. Acad. Sci. U S A *101*, 4673–4678.

Cargill, M., Schrodi, S.J., Chang, M., Garcia, V.E., Brandon, R., Callis, K.P., Matsunami, N., Ardlie, K.G., Civello, D., Catanese, J.J., et al. (2007). A large-scale genetic association study confirms IL12B and leads to the identification of IL23R as psoriasis-risk genes. Am. J. Hum. Genet. *80*, 273–290.

Chen, Y., Müller, J.D., So, P.T.C., and Gratton, E. (1999). The photon counting histogram in fluorescence fluctuation spectroscopy. Biophys. J. *77*, 553–567.

Cheng, Y.-C., and Prusoff, W.H. (1973). Relationship between the inhibition constant (KI) and the concentration of inhibitor which causes 50 per cent inhibition (I50) of an enzymatic reaction. Biochem. Pharmacol. *22*, 3099–3108.

Cheng, X., Lee, T.-Y., Ledet, G., Zemade, G., Tovera, J., Campbell, R., Purro, N., Annamalai, T., Masjedizadeh, M., Liu, D., et al. (2019). Safety, tolerability, and pharmacokinetics of PTG-200, an oral GI-restricted peptide antagonist of IL-23 receptor, in normal healthy volunteers. Am. J. Gastroenterol. *114*, 439–440.

Chua, A.O., Chizzonite, R., Desai, B.B., Truitt, T.P., Nunes, P., Minetti, L.J., Warrier, R.R., Presky, D.H., Levine, J.F., Gately, M.K., et al. (1994). Expression cloning of a human IL-12 receptor component: a new member of the cytokine receptor superfamily with strong homology to gp130. J. Immunol. *153*, 128–136.

Chyuan, I.T., and Lai, J.H. (2020). New insights into the IL-12 and IL-23: from a molecular basis to clinical application in immune-mediated inflammation and cancers. Biochem. Pharmacol. *175*, 113928.

Cochet, C., Kashles, O., Chambaz, E.M., Borrello, I., King, C.R., and Schlessinger, J. (1988). Demonstration of epidermal growth factor-induced receptor dimerization in living cells using a chemical covalent cross-linking agent. J. Biol. Chem. *263*, 3290–3295.

Couturier, C., and Jockers, R. (2003). Activation of the leptin receptor by a ligand-induced conformational change of constitutive receptor dimers. J. Biol. Chem. *278*, 26604–26611.

Crivat, G., and Taraska, J.W. (2012). Imaging proteins inside cells with fluorescent tags. Trends Biotechnol. *30*, 8–16.

Cua, D.J., Sherlock, J., Chen, Y., Murphy, C.A., Joyce, B., Seymour, B., Lucian, L., To, W., Kwan, S., Churakova, T., et al. (2003). Interleukin-23 rather than interleukin-12 is the critical cytokine for autoimmune inflammation of the brain. Nature *421*, 744–748.

Dacres, H., Michie, M., Wang, J., Pfleger, K.D.G., and Trowell, S.C. (2012). Effect of enhanced Renilla luciferase and fluorescent protein variants on the Förster distance of Bioluminescence resonance energy transfer (BRET). Biochem. Biophys. Res. Commun. *425*, 625–629.

Dong, H., Li, Q., Zhang, Y., Tan, W., and Jiang, Z. (2013). IL23R gene confers susceptibility to ankylosing spondylitis concomitant with uveitis in a Han Chinese population. PLoS One *8*, e67505.

Duerr, R.H., Taylor, K.D., Brant, S.R., Rioux, J.D., Silverberg, M.S., Daly, M.J., Steinhart, A.H., Abraham, C., Regueiro, M., Griffiths, A., et al. (2006). A genome-wide association study identifies IL23R as an inflammatory bowel disease gene. Science *314*, 1461–1463.

Engelowski, E., Schneider, A., Franke, M., Xu, H., Clemen, R., Lang, A., Baran, P., Binsch, C., Knebel, B., Al-Hasani, H., et al. (2018). Synthetic cytokine receptors transmit biological signals using artificial ligands. Nat. Commun. *9*, 2034.

Esch, A., Masiarz, A., Mossner, S., Moll, J.M., Grötzinger, J., Schröder, J., Scheller, J., and Floss, D.M. (2020). Deciphering site 3 interactions of interleukin 12 and interleukin 23 with their cognate murine and human receptors. J. Biol. Chem. *295*, 10478–10492.

Fassbender, K., Ragoschke, A., Rossol, S., Schwartz, A., Mielke, O., Paulig, A., and Hennerici, M. (1998). Increased release of interleukin-12p40 in MS: association with intracerebral inflammation. Neurology *51*, 753–758.

Floss, D.M., Moll, J.M., and Scheller, J. (2020). IL-12 and IL-23—close relatives with structural homologies but distinct immunological functions. Cells *9*, 2184.

Gaffen, S.L., Jain, R., Garg, A.V., and Cua, D.J. (2014). The IL-23-IL-17 immune axis: from mechanisms to therapeutic testing. Nat. Rev. Immunol. *14*, 585–600.

Gent, J., Van Kerkhof, P., Roza, M., Bu, G., and Strous, G.J. (2002). Ligand-independent growth hormone receptor dimerization occurs in the endoplasmic reticulum and is required for ubiquitin system-dependent endocytosis. Proc. Natl. Acad. Sci. U S A *99*, 9858–9863.

Gherbi, K., May, L.T., Baker, J.G., Briddon, S.J., and Hill, S.J. (2015). Negative cooperativity across β1-adrenoceptor homodimers provides insights into the nature of the secondary low-affinity CGP 12177 β1-adrenoceptor binding conformation. FASEB J. *29*, 2859–2871.

Glassman, C.R., Mathiharan, Y.K., Jude, K.M., Su, L., Panova, O., Lupardus, P.J., Spangler, J.B., Ely, L.K., Thomas, C., Skiniotis, G., et al. (2021). Structural basis for IL-12 and IL-23 receptor sharing reveals a gateway for shaping actions on T versus NK cells. Cell *184*, 983–999.e24.

Hasegawa, H., Mizoguchi, I., Chiba, Y., Ohashi, M., Xu, M., and Yoshimoto, T. (2016). Expanding diversity in molecular structures and functions of the IL-6/IL-12 heterodimeric cytokine family. Front. Immunol. *7*, 479.

Hummel, T.M., Ackfeld, T., Schönberg, M., Ciupka, G., Schulz, F., Oberdoerster, A., Grötzinger, J., Scheller, J., and Floss, D.M. (2017). Synthetic deletion of the interleukin 23 receptor (IL-23R) stalk region led to autonomous IL-23R homodimerization and activation. Mol. Cell. Biol. *37*, e00014–e00017.

Jullien, D., Prinz, J.C., and Nestle, F.O. (2015). Immunogenicity of biotherapy used in psoriasis: the science behind the scenes. J. Invest. Dermatol. *135*, 31–38.

Kilpatrick, L.E., Friedman-Ohana, R., Alcobia, D.C., Riching, K., Peach, C.J., Wheal, A.J., Briddon, S.J., Robers, M.B., Zimmerman, K., Machleidt, T., et al. (2017). Real-time analysis of the binding of fluorescent VEGF165a to VEGFR2 in living cells: effect of receptor tyrosine kinase inhibitors and fate of internalized agonist-receptor complexes. Biochem. Pharmacol. *136*, 62–75.

Kong, X.D., Moriya, J., Carle, V., Pojer, F., Abriata, L.A., Deyle, K., and Heinis, C. (2020). De novo development of proteolytically resistant therapeutic peptides for oral administration. Nat. Biomed. Eng. *4*, 560–571.

Kuchař, M., Vaňková, L., Petroková, H., Černý, J., Osička, R., Pelák, O., Šípová, H., Schneider, B., Homola, J., Šebo, P., et al. (2014). Human interleukin-23 receptor antagonists derived from an albumin-binding domain scaffold inhibit IL-23-dependent ex vivo expansion of IL-17-producing T-cells. Proteins *82*, 975–989.

Langrish, C.L., Chen, Y., Blumenschein, W.M., Mattson, J., Basham, B., Sedgwick, J.D., McClanahan, T., Kastelein, R.A., and Cua, D.J. (2005). IL-23 drives a pathogenic T cell population that induces autoimmune inflammation. J. Exp. Med. *201*, 233–240.

Machleidt, T., Woodroofe, C.C., Schwinn, M.K., Méndez, J., Robers, M.B., Zimmerman, K., Otto, P., Daniels, D.L., Kirkland, T.A., and Wood, K.V. (2015). NanoBRET—a novel BRET platform for the analysis of protein-protein interactions. ACS Chem. Biol. *10*, 1797–1804.

Maruyama, I.N. (2015). Activation of transmembrane cell-surface receptors via a common mechanism? The "rotation model". BioEssays *37*, 959–967.

Ngiow, S.F., Teng, M.W.L.L., and Smyth, M.J. (2013). A balance of interleukin-12 and -23 in cancer. Trends Immunol. *58*, 1208–1216.

Oppmann, B., Lesley, R., Blom, B., Timans, J.C., Xu, Y., Hunte, B., Vega, F., Yu, N., Wang, J., Singh, K., et al. (2000). Novel p19 protein engages IL-12p40 to form a cytokine, IL-23, with biological activities similar as well as distinct from IL-12. Immunity *13*, 715–725.

Pandya, P., Sayers, R.O., Ting, J.P., Morshedian, S., Torres, C., Cudal, J.S., Zhang, K., Fitchett, J.R., Zhang, Q., Zhang, F.F., et al. (2020). Integration of phage and yeast display platforms: a reliable and cost effective approach for binning of peptides as displayed on-phage. PLoS One *15*, e0233961.

Parham, C., Chirica, M., Timans, J., Vaisberg, E., Travis, M., Cheung, J., Pflanz, S., Zhang, R., Singh, K.P., Vega, F., et al. (2002). A receptor for the heterodimeric cytokine IL-23 is composed of IL-12R 1 and a novel cytokine receptor subunit, IL-23R. J. Immunol. *168*, 5699–5708.

Perpiñá-Viciano, C., Işbilir, A., Zarca, A., Caspar, B., Kilpatrick, L.E., Hill, S.J., Smit, M.J., Lohse, M.J., and Hoffmann, C. (2020). Kinetic analysis of the early signaling steps of the human chemokine receptor CXCR4. Mol. Pharmacol. *98*, 72–87.

Quiniou, C., Domínguez-Punaro, M., Cloutier, F., Erfani, A., Ennaciri, J., Sivanesan, D., Sanchez, M., Chognard, G., Hou, X., Rivera, J.C., et al. (2014). Specific targeting of the IL-23 receptor, using a novel small peptide noncompetitive antagonist, decreases the inflammatory response. Am. J. Physiol. Integr. Comp. Physiol. *82*, 1216–1230.

Remy, I., Wilson, I.A., and Michnick, S.W. (1999). Erythropoietin receptor activation by a ligand-induced conformation change. Science *283*, 990–993.

Schroder, J., Moll, J.M., Baran, P., Grotzinger, J., Scheller, J., and Floss, D.M. (2015). Non-canonical interleukin 23 receptor complex assembly: P40 protein recruits interleukin 12 receptor β1 via site II and induces P19/interleukin 23 receptor interaction via site III. J. Biol. Chem. *290*, 359–370.

Schuster, B., Meinert, W., Rose-John, S., and Kallen, K.J. (2003). The human interleukin-6 (IL-6) receptor exists as a preformed dimer in the plasma membrane. FEBS Lett. *538*, 113–116.

Shigehara, K., Shijubo, N., Ohmichi, M., Kamiguchi, K., Takahashi, R., Morita-Ichimura, S., Ohchi, T., Tatsuno, T., Hiraga, Y., Abe, S., et al. (2003). Increased circulating interleukin-12 (IL-12) p40 in pulmonary sarcoidosis. Clin. Exp. Immunol. *132*, 152–157.

Sivanesan, D., Beauchamp, C., Quinou, C., Lee, J., Lesage, S., Chemtob, S., Rioux, J.D., and Michnick, S.W. (2016). IL23R (interleukin 23 receptor) variants

CellPress
OPEN ACCESS

protective against inflammatory bowel diseases (IBD) display loss of function due to impaired protein stability and intracellular trafficking. J. Biol. Chem. *291*, 8673–8685.

Stoddart, L.A., Johnson, E.K.M., Wheal, A.J., Goulding, J., Robers, M.B., Machleidt, T., Woods, K.V., Hill, S.J., and Pfleger, K.D.G. (2015). Application of BRET to monitor ligand binding to GPCRs. Nature Method *12*, 661–663. https://doi.org/10.1038/nmeth.3398.

Tait Wojno, E.D., Hunter, C.A., and Stumhofer, J.S. (2019). The immunobiology of the interleukin-12 family: room for discovery. Immunity *50*, 851–870.

Varghese, T.M., Dudas, P.L., Allen, S.J., Schneeweis, J.E., and Finley, M.F.A. (2020). Optimization of a high-throughput cell-based screening strategy to identify small-molecule inhibitors of IL-23 signaling. SLAS Discov *26*, 122–129. https://doi.org/10.1177/2472555220923362.

Verreck, F.A.W., De Boer, T., Langenberg, D.M.L., Hoeve, M.A., Kramer, M., Vaisberg, E., Kastelein, R., Kolk, A., De Waal-Malefyt, R., and Ottenhoff, T.H.M. (2004). Human IL-23-producing type 1 macrophages promote but IL-10-producing type 2 macrophages subvert immunity to (myco)bacteria. Proc. Natl. Acad. Sci. U S A *101*, 4560–4565.

Vignali, D.A.A., and Kuchroo, V.K. (2012). IL-12 family cytokines: immunological playmakers. Nat. Immunol. *13*, 722.

Wang, R.X., Yu, C.R., Mahdi, R.M., and Egwuagu, C.E. (2012). Novel IL27p28/IL12p40 cytokine suppressed experimental autoimmune uveitis by inhibiting autoreactive Th1/Th17 cells and promoting expansion of regulatory T cells. J. Biol. Chem. *287*, 36012–36021.

Waters, M.J., Brooks, A.J., and Chhabra, Y. (2014). A new mechanism for growth hormone receptor activation of JAK2, and implications for related cytokine receptors. JAK-STAT *3*, 2162–3996.

Werner, J.L., Gessner, M.A., Lilly, L.M., Nelson, M.P., Metz, A.E., Horn, D., Dunaway, C.W., Deshane, J., Chaplin, D.D., Weaver, C.T., et al. (2011). Neutrophils produce interleukin 17A (IL-17A) in a Dectin-1- and IL-23-dependent manner during invasive fungal infection. Infect. Immun. *79*, 3966–3977.

Ye, J., Wang, Y., Wang, Z., Liu, L., Yang, Z., Wang, M., Xu, Y., Ye, D., Zhang, J., Lin, Y., et al. (2020). Roles and mechanisms of interleukin-12 family members in cardiovascular diseases: opportunities and challenges. Front. Pharmacol. *11*, 129.

STAR★METHODS

KEY RESOURCES TABLE

REAGENT or RESOURCE	SOURCE	IDENTIFIER
Antibodies		
Rabbit Anti-NanoLuciferase	Promega Corporation	Gifted by Promega
AF488 Chicken Anti-Rabbit	ThermoFisher Scientific	Cat# A-21441; RRID: AB_2535859
Chemicals, peptides, recombinant proteins		
HaloTag NanoBRET 618 Ligand	Promega Corporation	Cat# G9801
SNAP-tag AlexaFluor 488 membrane impermeant substrate	New England BioLabs	Cat# S9124S
AlexaFluor 488 HaloTag Ligand	Promega Corporation	Cat# G1001
5(6)-TAMRA (5-(and-6)-Carboxytetramethylrhodamine), mixed isomers	ThermoFisher Scientific	Cat# C300
TAMRA, SE; 5-(and-6)-Carboxytetramethylrhodamine, Succinimidyl Ester (5(6)-TAMRA, SE), mixed isomers	ThermoFisher Scientific	Cat# C1171
Recombinant IL-23 protein	GlaxoSmithKline (internal)	Gifted by Surjit Bains
FuGENE HD	Promega	Cat# E2312
Fetal Bovine Serum	Sigma Aldrich	Cat# F2442
Protease-free Bovine Serum Albumin	Sigma Aldrich	Cat# A7030
Dulbecco’s Modified Eagle’s Medium	Sigma Aldrich	Cat# D6429
Poly-D-Lysine hydrobromide	Sigma Aldrich	Cat# P6407
Phosphate Buffered Saline (PBS)	Sigma Aldrich	Cat# D8537
Opti-MEM reduced serum medium	ThermoFisher Scientific	Cat# 11058021
Dithiothreitol (DTT)	ThermoFisher Scientific	Cat# R0862
Formic acid	Fisher Chemical	Cat# F/1900/PB15
Acetonitrile	Sigma-Aldrich	Cat# 34851
Paraformaldehyde (PFA)	Sigma Aldrich	Cat# F8775
Glycine	Sigma Aldrich	Cat# G8898
Chicken Serum	Sigma Aldrich	Cat# C5405
Immersol 518F (30°C) oil	Zeiss	Cat# 444970-9000-000
Hoechst Nuclear Stain (H33342)	Sigma Aldrich	Cat# B2261
Purified NanoLuciferase	Veprintsev Lab	Gifted by Bradley Hoare
Critical commercial assays		
Nano-Glo luciferase assay system (Furimizine)	Promega Corporation	Cat# N1130
AlphaLISA SureFire Ultra p-STAT3 (Tyr705) Assay Kit	Perkin Elmer	Cat# ALSU-PST3
Experimental models: Cell lines		
Human: HEK293T cells (female)	ATCC (Virginia, USA)	Cat# CRL-3216
Recombinant DNA		
IL6SS-NL-IL12Rβ1	Genscript	Custom synthesis
IL6SS-NL-IL23R	Genscript	Custom synthesis
IL6SS-HT-TEV-IL12Rβ1	Genscript	Custom synthesis
IL6SS-HT-TEV-IL23R	Genscript	Custom synthesis
IL23R-MycDDK expression plasmid	Origene	Cat# RC211477
IL12Rβ1 expression plasmid	Origene	Cat# SC303661

(Continued on next page)

Continued

REAGENT or RESOURCE	SOURCE	IDENTIFIER
pc 3.1 zeo SigSNAP-tag	(Gherbi et al., 2015)	Custom synthesis
pc3.1 zeo	Invitrogen	Cat# V86020
Software and algorithims		
GraphPad Prism 7.02	GraphPad Software	www.graphpad.com
Zen 2012	Zeiss	www.zeiss.com
MaxEnt1 4.1	Waters	www.waters.com
ImageJ Fiji 1.53	National Institute of Health	www.fiji.sc
Other		
White 96-well plates	Greiner Bio-One	Cat# 655098
Poroshell 300SB-C3 column	Agilent	Cat# PN 821075-924
PD10 column	Cytiva	Cat# 17085101
Nunc Lab-Tek 8-well chambered coverslips	ThermoFisher Scientific	Cat# 1554411
35 mm glass bottom dish	MatTek	Cat# P35G-1.5-14-C
384 well white Optiplate	Perkin Elmer	Cat# 6007290
Xba1 restriction enzyme	Promega Corporation	Cat# R6181
Xho1 restriction enzyme	Promega Corporation	Cat# R6161
T4 Ligase	New England BioLabs	Cat# M0202S
DAM negative *E. coli*	Agilent Technologies	Cat# 200247
DH5 alpha *E. coli*	Invitrogen	Cat# 18265017

RESOURCE AVAILABILITY

Lead contact

Requests for resource and reagent sharing should be directed to and will be fulfilled by the lead contact, Professor Stephen J Hill (Stephen.hill@nottingham.ac.uk).

Materials availability

All unique/stable reagents generated in this study are available from the Lead Contact with a completed Materials Transfer Agreement.

Data and code availability

This study did not generate/analyze any computational datasets/code.

EXPERIMENTAL MODEL AND SUBJECT DETAILS

Female HEK293T cells were cultured and transfected as described in Method details.

METHOD DETAILS

Materials

All reagents unless otherwise stated were purchased from Sigma-Aldrich. Xba1 and Xho1 restriction enzymes, pNLF and FuGENE HD transfection reagent, pNLF vectors, NanoGlo Substrate, AF488 HaloTag Ligand and HaloTag NanoBRET 618 Ligand were purchased from Promega. OptiMEM and NHS Ester linked 5 and 6-Carboxytetramethylrhodamine (TAMRA) mixed isomers were purchased from Thermo-Fisher Scientific. SNAP-Surface Alexa Fluor 488 was purchased from New England BioLabs. An IL23R-MYCDDK expression plasmid (accession code NM_144701) and an IL12Rβ1 expression plasmid (accession code NM_005535) were purchased from Origene. Recombinant NanoLuciferase expressed in E. coli and purified using an N terminal His-tag was gifted by Bradley Hoare of the Veprintsev lab, University of Nottingham. Rabbit anti-Nanoluciferase was kindly gifted by Promega. Recombinant IL-23 protein was gifted by Surjit Bains at GlaxoSmithKline (Stevenage, UK). The protein was originally purified through expression in HEK293-F cells via co-transduction with BacMam viruses containing the p19-His and p40 transcripts. Heterodimeric IL-23 was then purified from the supernatant using a Ni-Sepharose column.

Molecular biology

Construction of N-terminal fusion constructs

NL constructs were custom made at Genscript by synthesis of either IL23R or IL12Rβ1 without their endogenous signal peptides and the subsequent sub-cloning of these, into a pNLF vector (Promega) using Xho1 and Xba1, which previously had an IL-6 secretion signal added to the N terminus of NanoLuc via mutagenesis. The resulting open reading frames encoded an *N*-terminal IL-6 secretion signal followed by NL then a Gly-Ser-Arg linker between the NL fusion and the N terminus of either IL23R or IL12Rβ1. HaloTag constructs were custom synthesised at Genscript by synthesis of cDNA encoding an *N*-terminal IL-6 secretion signal followed by HaloTag which was fused to either IL23R or IL12Rβ1 by a Glu-Pro-Thr-Thr-Glu-Asp-Leu-Tyr-Phe-Gln-Ser-Asp-Asn linker that contained a Tobacco Etch Virus (TEV) protease site. These cDNAs were then sub cloned into pNLF plasmids via Nhe1 and Not1 restriction sites.

N-terminal SNAP-tag constructs were created by sub cloning IL23R and IL12Rβ1 genes from the previously outlined *N*-terminal NanoLuc fusion plasmids (grown in DAM negative *E. coli*) into a pc3.1 zeocin vector with and *N*-terminal SNAP-tag fused with a murine 5-HT3a receptor signal sequence, that has previously been described (Gherbi et al., 2015), using the restriction sites Xho1 and Xba1. This resulted in a linker between SNAP-tag and the gene with a sequence of Ser-Thr-Ser-Pro-Val-Trp-Trp-Asn-Ser-Ala-Asp-Ile-Gln-His-Ser-Gly-Gly-Arg-Ser-Arg.

Labelling of purified IL-23 protein

Recombinant IL-23 was labelled by incubation of 100 μg of protein with a 3 times molar ratio (21.4 μM) of NHS ester coupled TAMRA dye, at room temperature for 2 hours in a pH 7.4 Phosphate Buffered Saline (PBS) buffer. The labelled protein was separated from the reaction mixture through elution in a PD10 desalting column (Cytiva). Absorbance of the fractions at both 280 and 557 nm was then quantified through the use of a Nanodrop Spectrophotometer (ThermoFisher Scientific) and combined into aliquots of varying IL-23 concentrations (as defined by 280 nm absorbance).

LC-MS analysis of TAMRA labelled IL-23 cytokine

Unfolding intact mass experiments were carried out using liquid chromatography coupled mass spectrometry (LC-MS). Samples were analysed on Reversed-Phase (RP) chromatography (BioResolve RP column (2.1 x 50 mm, 2.7 μM, PN: on BioAccord RDa system (Waters). Samples were desalted by washing with 0.1% formic acid in 25% acetonitrile and eluted using linear gradient with 0.1% formic acid up to 80% acetonitrile at a flow rate of 0.5 ml/min. The divert valve was used and directed flow to waste from 0 to 0.5 min after injection to avoid source contamination with buffer components. The eluate was ionised by electrospray ionisation (ESI). The column temperature was maintained at 80°C, RDa acquisition was set to high mass range (400-7000 *m/z*) in positive ion mode with the following source settings: cone voltage - 70 V, capillary voltage - 1.5 kV and the desolvation temperature - 550°C.

Non-reduced samples were injected into LC-MS directly from the stock buffer, when samples were run reduced, 50 mM final concentration of DTT was added to sample prior to injection. Chromatography peaks were integrated between 0.5 and 1.5 min and mass spectra were deconvolved using the MaxEnt1 algorithm.

Fluorescence correlation spectroscopy analysis of IL23-TMR

Solution Fluorescence Correlation Spectroscopy (FCS) was carried out as previously described (Briddon et al., 2004; Kilpatrick et al., 2017). Briefly samples were imaged in 8 well chambered Nunc Labtek coverglasses (No. 1.0 borosilicate glass bottom; ThermoFisher Scientific) using a Zeiss LSM 880 microscope with a 40X c-Apochromat 1.2 NA water-immersion objective (Zeiss) at 24°C. Samples were excited with a Diode pumped solid state (DPSS) 561 nm laser and emission light collected through a 553-695 nm band pass onto a GaAsP detector using a pinhole set at 1 airy unit. The confocal volume was set to 200 μm above the coverslip surface and beam paths were calibrated using a solution of 20 nM TAMRA (5-6 carboxy mixed isomers; D = 2.88×10^{-10} m^2/s;) prepared in high performance liquid chromatography grade water (Chromasolv). Calibration measurements were collected using ten 10 s and a one 60 s reads. FCS measurements were performed using a range of IL23-TMR concentrations in PBS with or without 1 mg/ml protease-free BSA . Measurements were recorded at 1 kW/cm^2 laser power for four 15 s reads.

Cell culture

Human Embryonic Kidney 293T (HEK293T) cells were purchased from ATCC and cultured in Dulbecco's Modified Eagle Medium (DMEM) with 10% Fetal Bovine Serum (FBS) in tissue culture flasks at 37.5°C and 5% CO_2. All experimental incubations outlined in DMEM based media were carried out at 37.5°C and 5% CO_2 and incubations in Hanks Balanced Saline Solution (HBSS) based media were carried out 37.5°C without added CO_2.

Transient transfections

For all experiments except those where transfection conditions were varied to induce changes in intra-receptor NanoBRET, cells were transfected as follows; HEK293T cells were seeded in six well plates and incubated for 4-6 hours followed by addition of 100 μl of a transfection mixture consisting of FuGENE HD at a 3:1 ratio to DNA plasmid in OptiMEM. 2 μg per well total DNA concentration with 1 μg of NL fused receptor construct and either 1 μg of untagged partner receptor or pc3.1 zeocin plasmid to normalise DNA concentrations was used in all experiments except NL- IL12Rβ1 and IL23R binding experiments where a 1:10 ratio was used with 2.2 μg of total DNA per well cells were then incubated overnight.

In intra-receptor NanoBRET experiments with a range of transfection conditions HEK293T cells were adjusted to 150000 cells/ml and dispensed at 100 μl into a poly-d-lysine (PDL) coated 96 well white microplate. Cells were then incubated overnight before being

transfected with 50 ng well NL-IL23R and varying concentrations of SNAP-IL12Rβ1 made up to 100ng/well with pc3.1 zeocin vector and with 0.3 μl FuGENE per well. The transfection mixture was made up to 5 μl per well with OptiMEM. Cells were then incubated overnight.

For 8 well imaging experiments HEK293T cells were seeded at 200000 cells per well in 300 μl of media into PDL coated 8 well glass plates. The following day each well was transfected with a 10 μl transfection mix made up of 200 ng cDNA consisting of 100 ng of each receptor construct and 0.6 μl of FuGENE HD in OptiMEM. 100 ng of pc3.1 zeocin empty vector was included if only one receptor monomer was to be expressed.

Luminescence imaging

HEK293T cells were transfected as described in the transient transfection section except for their initial seeding into PDL coated 35mm glass bottom plates (Matek) at a density of 150000 cells/ml in 2 ml of DMEM with 10% FBS. After transfection the dishes were incubated for 2 days before media was replaced with 3 ml of 37°C HBSS with 5.13 μM Furimizine. The dishes were then imaged on an LV200 luminescence microscope (Olympus) equipped with a C9100-23B IMAGE EMX2 camera (Hamamatsu) in both bright-field and luminescence settings using a 60x/1.42NA oil immersion objective lens and 0.5x tube lens. Exposure was adjusted to the level of expression (2-15 s).

Immuno-cytochemical imaging

HEK293T cells were seeded and transfected in 8 well glass bottom plates as described in the transient transfection section. The following day cells were washed with PBS and fixed with 3% Paraformaldehyde (PFA) for 15 minutes. The cells were then washed three times with PBS and incubated with 30 mg/ml BSA and 10 mg/ml Glycine in PBS for 30 minutes. The cells were washed three times with PBS and then incubated in 10% chicken serum in PBS for 30 minutes. This mixture was then replaced with 1000 fold diluted rabbit anti-NanoLuciferase antibody in PBS with 10% chicken serum. The plates were then incubated overnight at 4°C. The following day the cells were washed three times with PBS and a 500 fold dilution of AF488 labelled chicken anti-rabbit antibody (ThermoFisher Scientific) in PBS with 10% chicken serum was added. Following an hour long incubation, cells were washed three times with PBS and 2 mg/ml Hoechst nuclear stain in PBS was added. The plates were incubated for 10 minutes, washed three times with PBS and then imaged using a Zeiss LSM 880 microscope fitted with a 63x PlanAprochromat oil objective (1.4 NA, Zeiss), using an Argon 488 nm laser (2%) and a 405-30 nm diode laser (2%) for excitation. The pinhole was set at 1 Airy Unit and AF488 and Hoescht imaged on separate tracks using a 488 nm beamsplitter and 495-630 nm band pass or a 405 nm beamsplitter and a 410-495 nm band pass respectively. All images were taken with 1024 x1024 pixels per frame with 8 averages.

NanoBRET IL23-TMR ligand binding and competition experiments

Transfected HEK293T cells were incubated overnight before being re-suspended, adjusted to 200000 cells/ml and added to PDL coated 96 well white clear bottom assay microplates (Griener Bio-One) at 100 μl per well. The plates were then incubated overnight before media was removed the following day and replaced with 50 μl per well of IL23-TMR in HBSS with 1 mg/ml BSA either with or without an excess unlabelled IL-23 to quantify non-specific binding, in ligand binding experiments. The concentration used depended on the potency of the interaction, 50 nM was used in NL-IL23R and IL12Rβ1 expression experiments, 100 nM in NL-IL12Rβ1 and IL23R experiments and 1 μM in NL-IL23R or NL-IL12Rβ1 experiments. In competition experiments media was replaced with 50 μl per well of a concentration titration of IL-23 in HBSS with 1 mg/ml BSA and differing concentrations of IL23-TMR. Plates were then incubated for 1 hour before the addition of 5 μl of 77 μM Furimizine per well a further 2 minute incubation before reading the plate on a Pherastar FS plate reader (BMG labtech) using a 450 nm (30 nm bandpass) and >550 nm filter with gains of 2000 and 3000 respectively.

Intra-receptor NanoBRET

To measure agonist induced changes in intra-receptor NanoBRET with a titration of IL-23, HEK293T cells that had previously been transfected were re-suspended and adjusted to 200000 cells/ml. Cells that expressed HaloTag fused constructs then had 100 nM HaloTag 618 ligand added. All cells were then dispensed at 100 μl into PDL coated 96 well white clear bottom microplates.

On the day of the experimental read cells transfected with SNAP-tag fused constructs had media replaced with 50 μl of DMEM with FBS with 0.2 μM SNAP-tag-AF488 membrane impermeant substrate and were then incubated for 30 minutes followed by three washes with HBSS. The buffer for the labelled cells was then replaced with HBSS with 1 mg/ml BSA with or without varying concentrations of IL-23 and incubated for one hour. 5 μl of 77 μM Furimizine per well was then added and a further 2 minute incubation before plates were read using a Pherastar FS. Cells labelled with SNAP-tag AF488 substrate

were read using 475 nm (30 nm bandpass) and 535 nm (30 nm bandpass) filters with gains of 2800 and 3600 respectively. Cells labelled with Halotag 618 dye were read using 460 nm (80 nm bandpass) and >610 nm longpass filters with gains of 2000 and 3000 respectively.

When a range of transfections was being used to measure constituitive association of IL-23 receptor subunits the protocol was performed as above except that cells were first read for fluorescent intensity before the addition of Furimizine using excitation at 485 nm and emission at 520 nm measured using a PheraStar FS.

Quantification of STAT3 phosphorylation

Transfected HEK293T cells were plated into a PDL coated 96 well tissue culture microplate and then incubated overnight. The following day media was replaced with serum free DMEM and the cells incubated for 3 hours. Media was then replaced with

HBSS containing 1 mg/ml BSA and a concentration titration of IL-23 and the cells incubated for 30 minutes. An AlphaLISA SureFire Ultra assay was then used to measure STAT3 phosphorylation at residue Tyr705. Assays were performed according to the manufacturers' instructions.

Imaging of SNAP-tag and HaloTag fused constructs

HEK293T cells were seeded and transfected in 8 well glass bottom plates as outlined in the transient transfection section. The following day cells were washed with PBS and then labelled with 500 nM of either AF488 HaloTag ligand or SNAP-tag AF488 substrate in HBSS and incubated for 30 minutes at 37°C. The cells were then washed three times with HBSS and fixed by incubation with 3% PFA in PBS for 15 minutes. The cells were then washed twice with PBS and then incubated with 2 mg/ml Hoechst stain in PBS for 10 minutes. The cells were then washed three times with PBS before being imaged on a Zeiss LSM 880 microscope as previously described in the immuno-cytochemical imaging section.

NL and SNAP-AF488 substrate standard curves

Serial dilutions of purified NL enzyme or SNAP-AF488 substrate were made up in 50 μl HBSS with 0.1% BSA in white clear bottom 96 well microplates. The plate containing the NL titration had 5 μl of 77 μM Furimizine added to each well and was then incubated at 37°C for 4 minutes. Both plates were then read on the same Pherastar plate reader used in cellular NanoBRET experiments, using the equivalent settings.

QUANTIFICATION AND STATISTICAL ANALYSIS

The LC-MS data was analysed using UNIFI v1.9.4 (Waters). Mass spectra of multiply charged state species were de-convoluted to produce mass output using the MaxEnt1 algorithm to the closest Da.

FCS analysis was carried out with Zen Black 2012 software (Zeiss). IL23-TMR autocorrelation data were fitted to a one-component, free 3D, Brownian diffusion model including a pre-exponential for triple state of the fluorophore (Kilpatrick et al., 2017). IL23-TMR data were also fitted to a two component photon counting histogram (PCH) model to derive the ratio of mono to multi-labelled particles. A 20 μs bin time was used for PCH analysis with the first order correction set to the value determined in the calibration measurements on each experimental day.

Scale bars were added to luminescence imaging data using Fiji version 1.53 (NIH).

Data were exported from a Pherastar plate reader as a BRET ratio, generated by dividing the acceptor signal at 535 ± 30, <550 or <610 nm by the donor signal at 475 ± 30, 450 ± 30 or 460 ± 80 nm respectively, depending on the filter used.

All further data analysis, including statistical tests, was carried out using Prism 7.02 software (GraphPad). Affinity measures for IL23-TMR were determined by fitting specific and non-specific binding to the equation:

$$BRET\ ratio = \frac{B_{max}[B]}{([B]+K_d)} + ((A[B]) + C\Big)$$

where B_{max} is the maximal specific binding, [B] is the concentration of IL23-TMR, K_d is the dissociation constant, A is the slope of the non-specific binding component and C is the Y intercept.

IL23-TMR binding comparison and ligand induced N terminal proximal change traces were generated by normalising the data to background and then fitting with the specific binding equation:

$$Y = \frac{B_{max}[B]}{([B]+K_d)}$$

where B_{max} is the maximal signal of the curve, [B] is the concentration of the ligand and K_d is the dissociation constant of the ligand. In some experiments the specific binding was fitted to a two-site binding equation:

$$Y = \frac{B_{max}1[B]}{([B]+K_d1)} + \frac{B_{max}2[B]}{([B]+K_d2)}$$

Where $B_{max}1$, $B_{max}2$, K_d1 and K_d2 are maximal specific binding levels and K_d values of the two components. Comparison of fits were made by analysis of the residual sum of squares using the partial F-test (GraphPad Prism).

Competition and STAT3 phosphorylation experiments were analysed by fitting the data with the 4 parameter equation:

$$Y = B_{min} + \frac{B_{max} - B_{min}}{1 + 10^{(LogXC50-[A])C}}$$

where B_{max} is the maximal signal, B_{min} is the minimum signal, LogXC50 is the log of the compound's 50% inhibitory concentration for competition experiments and 50% activating concentration in STAT3 reporter experiments, [A] is the concentration of competing drug and C is the Hill slope of the curve.

CellPress
OPEN ACCESS

$K_{i\ IL\text{-}23}$ values were generated from a mean of values generated from $IC_{50\ IL\text{-}23}$ measures by using the Cheng-Prusoff equation:

$$K_i = \frac{IC_{50}}{1 + \frac{[L]}{K_d}}$$

where K_d is the dissociation constant calculated for IL23-TMR previously in the ligand binding experiments and [L] is the concentration of IL23-TMR used.

Linear regression analysis was also performed on the relationship between IC_{50} and IL23-TMR concentration using a variant of the Cheng-Prusoff equation:

$$IC_{50} = [L]x\,\frac{K_i}{K_d} + K_i$$

Where a plot of IC_{50} versus [L] yielded a slope of K_i/K_d and the intercept provided K_i.

Cell Chemical Biology, Volume *29*

Supplemental information

Probing the binding of interleukin-23 to individual receptor components and the IL-23 heteromeric receptor complex in living cells using NanoBRET

Charles S. Lay, Angela Bridges, Joelle Goulding, Stephen J. Briddon, Zoja Soloviev, Peter D. Craggs, and Stephen J. Hill

Protein name	Predicted MW (Da)	Observed mass (Da)	Suspected PTMs
IL23p19-HisTag	19499.8	19499	none
IL12p40	34696.6	36555 (most abundant)	Mannose N-linked glycosylation
IL-23 complex	54181.2	56050 (most abundant)	Mannose N-linked glycosylation

Table S1: The masses of recombinant purified IL-23 subunits and heterodimeric complex used in the study. Predicted molecular weights were generated from the amino acid sequences of the proteins, assuming the reduction of internal disulphide bridges for the cytokine subunits. PTM, post-transaltional modification.

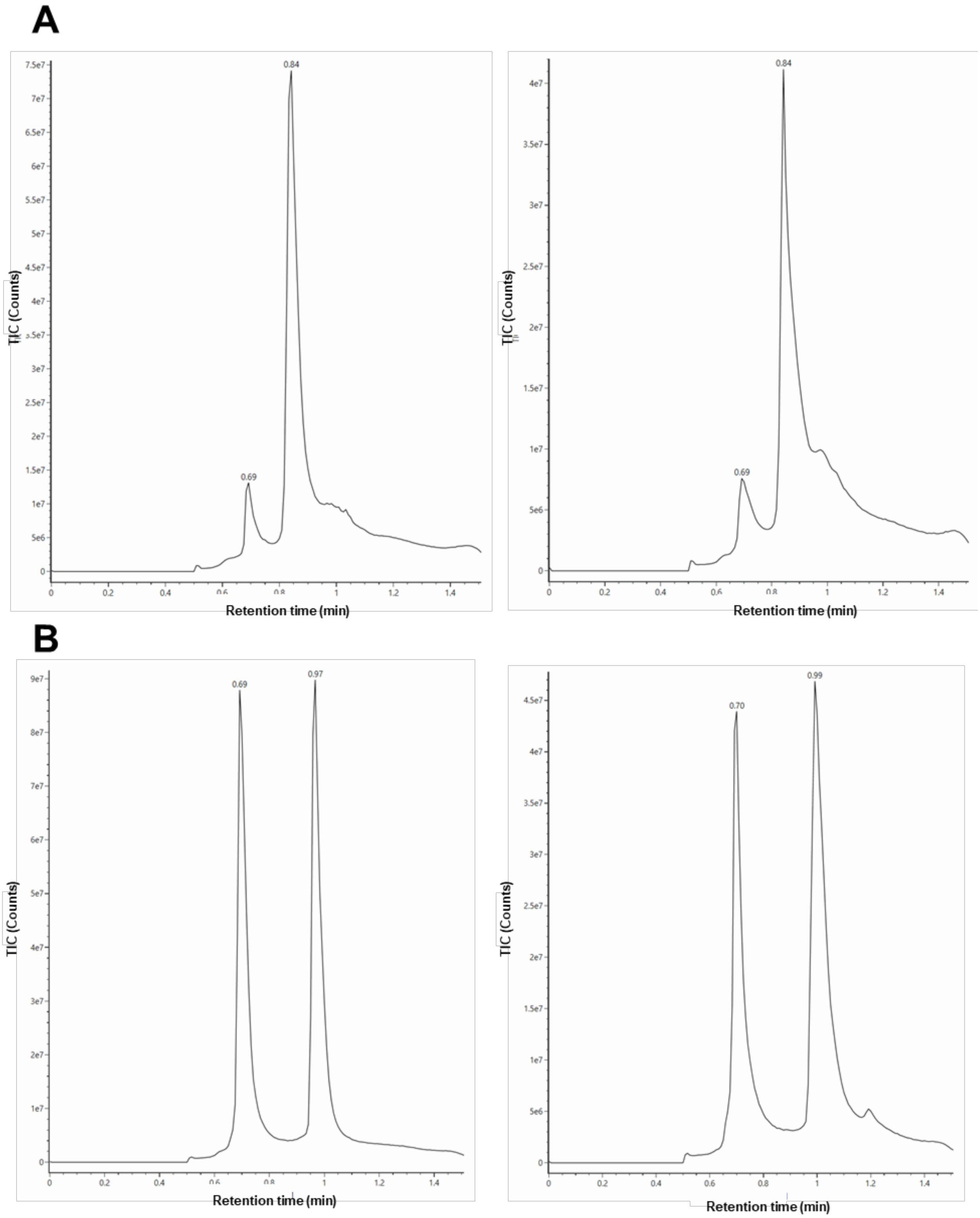

Figure S1 (Related to Figure 1): Reversed-Phase chromatography demonstrates heterodimeric IL-23 is reduced to its subunits by addition of DTT. (A) Total Ion Count (TIC) chromatograms of IL-23 (left) and IL23-TMR (right). (B) Total ion count chromatograms of IL-23 (left) and IL23-TMR (right), in the presence of 50 mM DTT.

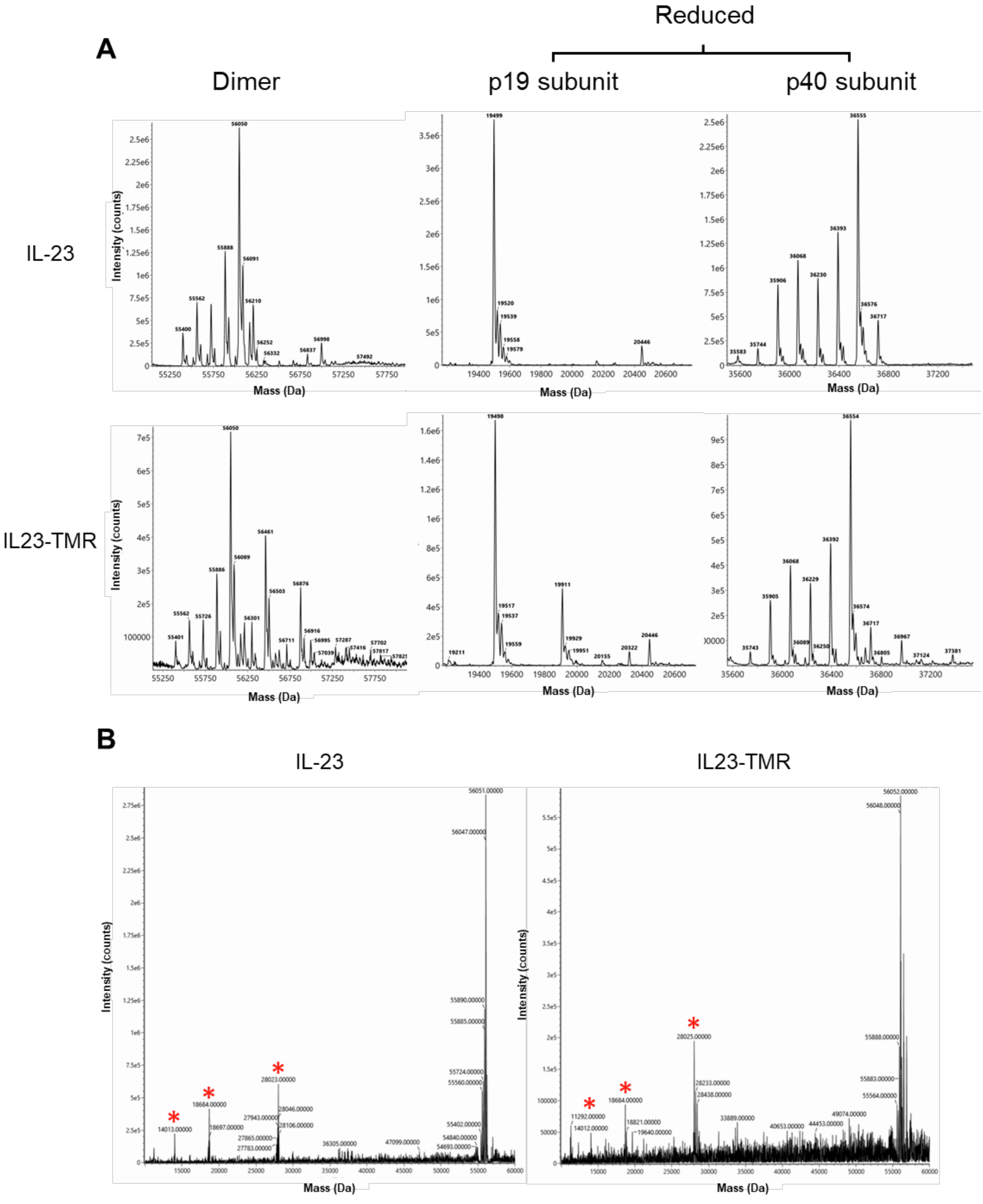
A
Reduced
Dimer
p19 subunit
p40 subunit
IL-23
IL23-TMR
Intensity (counts)
Mass (Da)
B
IL-23
IL23-TMR
Intensity (counts)
Mass (Da)

Figure S2 (Related to Figure 1): LC-MS analysis of IL23-TMR. (A) De-convolved LC-MS spectra of IL-23 (top) and IL23-TMR (bottom), demonstrating both the DTT reduced, p19 and p40 subunits (centre and right respectively) and the non-reduced dimeric cytokine (left). (B) De-convolved LC-MS spectra of IL-23 (left) and IL23-TMR (right) over a broad mass range. Resonance peak, deconvolution artefacts representing a half, third or fourth the mass of the true species are denoted by a red asterisk.

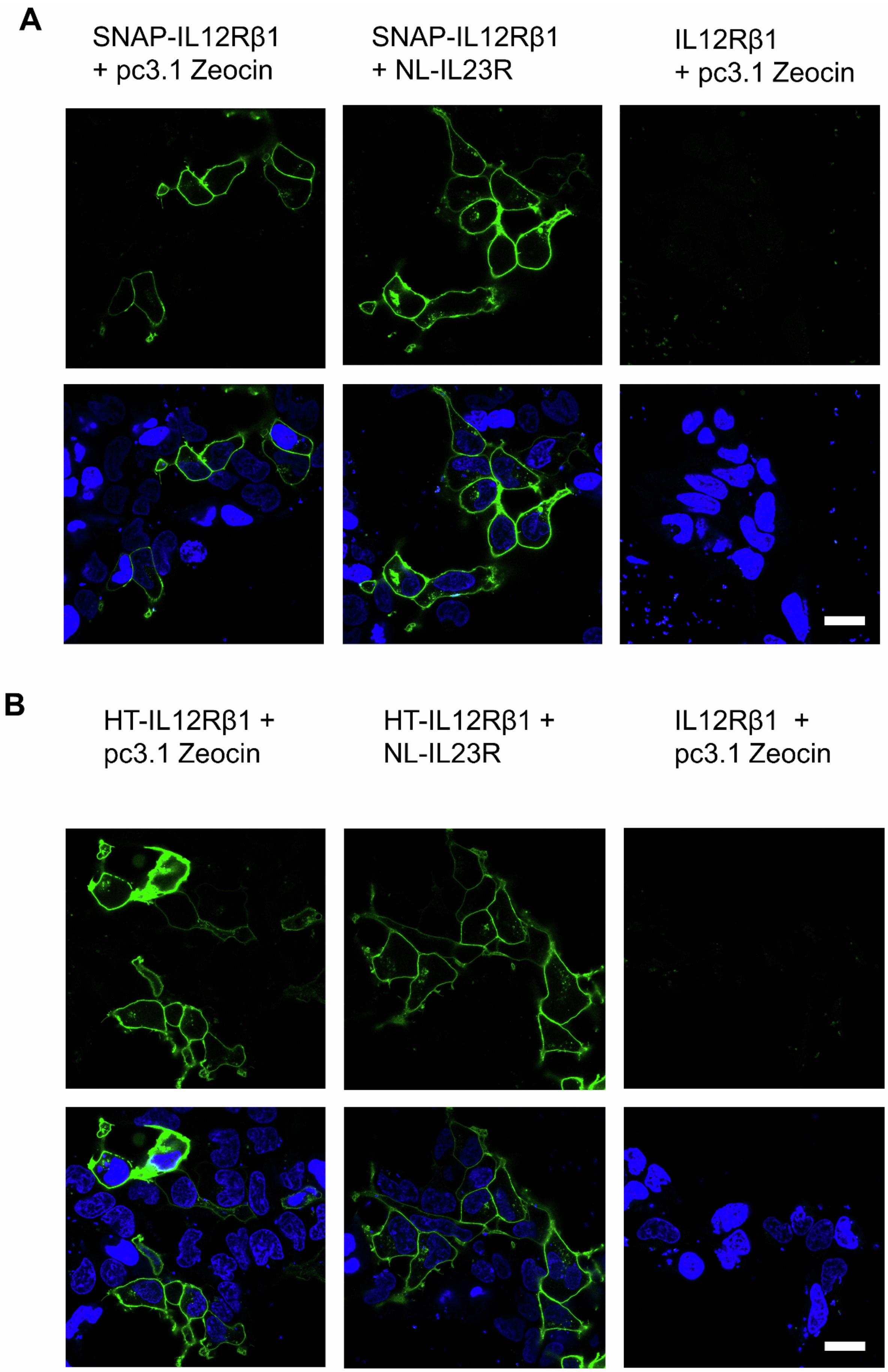
A
SNAP-IL12Rβ1
+ pc3.1 Zeocin
SNAP-IL12Rβ1
+ NL-IL23R
IL12Rβ1
+ pc3.1 Zeocin
B
HT-IL12Rβ1 +
pc3.1 Zeocin
HT-IL12Rβ1 +
NL-IL23R
IL12Rβ1 +
pc3.1 Zeocin

Figure S3 (Related to Figure 2): Surface expression of HaloTag and SNAP-Tag fused IL12Rβ1 constructs. (A) Transfected HEK293T cells labelled with SNAPTag-AF488 substrate with (bottom) and without (top) Hoerscht stain. Representative of 3 independent experiments. (B) Transfected HEK293T cells labelled with AF488 HaloTag Ligand with (bottom) and without (top) Hoerscht stain. Scale bars represent 20 μm. Images are representative of 3 independent experiments.

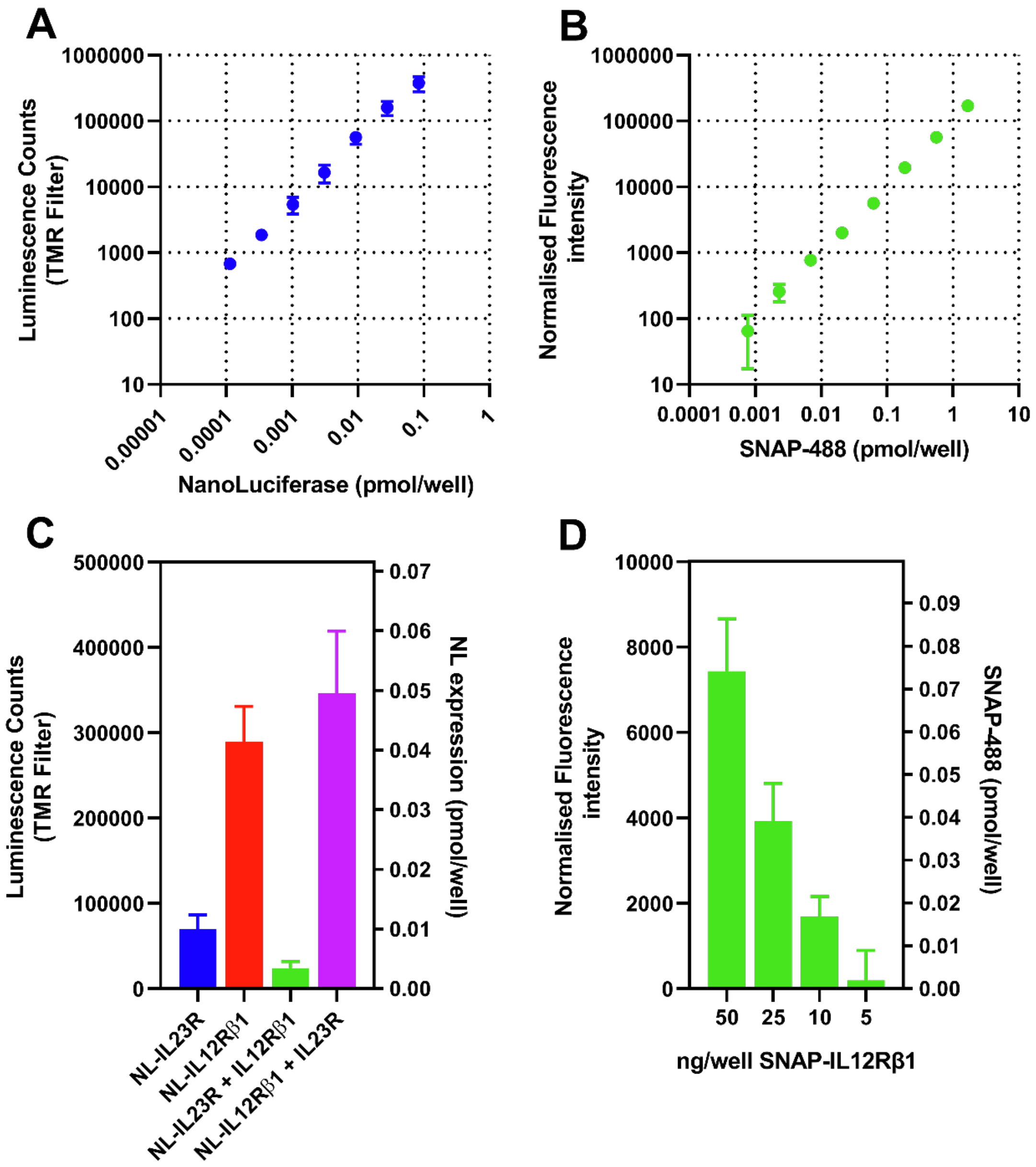

Figure S4 (Related to Figure 5 and Figure 7): Standard curves of purified NL and SNAP-AF488 allows the prediction of experimental construct concentration. (A) A standard curve of purified NL. Mean values with SEM from 3 independent experiments. (B) A standard curve of SNAP-AF488, normalised to background fluorescence intensity. Mean values with SEM from 4 independent experiments. (C) Transformation of luminescence values from HEK293T cells expressing equal quantities of NL-linked constructs to predicted NL expression values using the curve shown in (A). Transformation of fluorescent intensity values of cells transfected with varying concentrations of SNAP-IL12Rβ1 shown in Figure 7 to estimated SNAP-488 expression levels using the curve shown in (B).

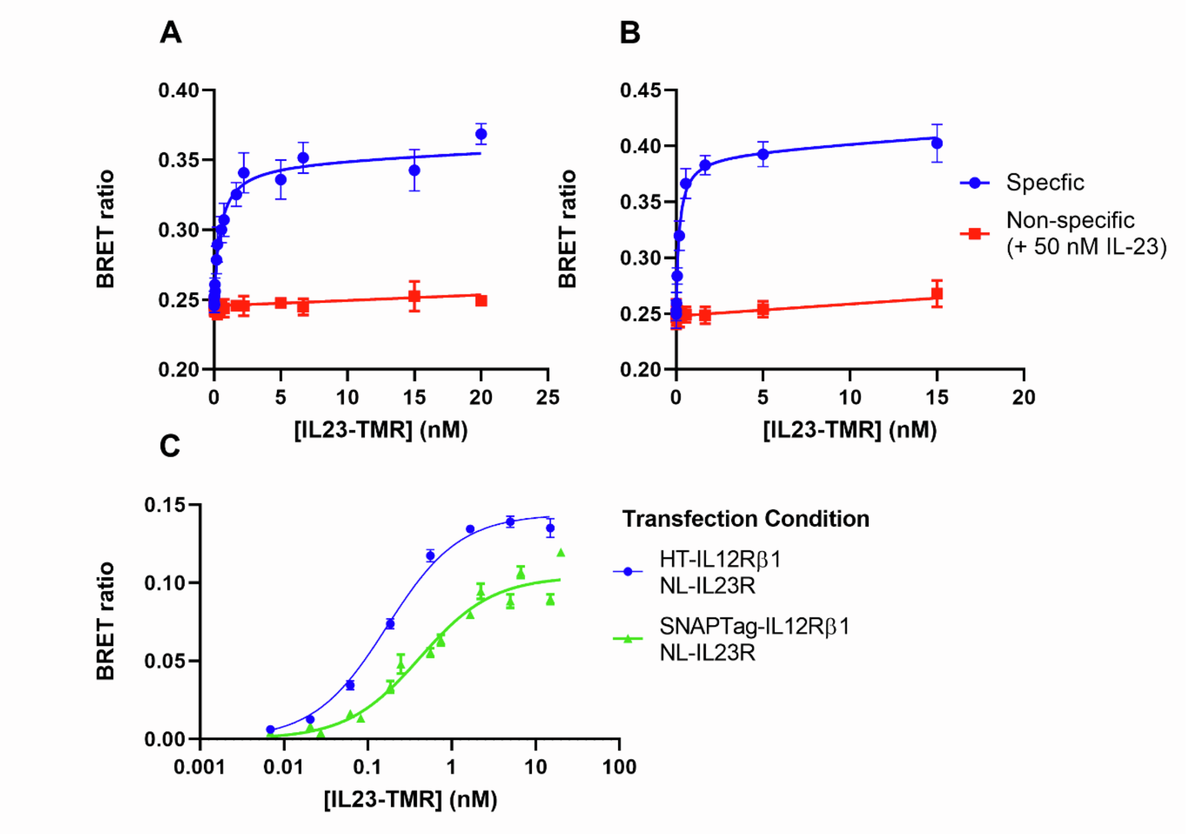

Figure S5 (Related to Figure 7): Fusion of SNAPTag or HaloTag to IL12Rβ1 does not block ligand binding. (A-B) The binding of IL23-TMR to cells expressing NL-IL23R and either HaloTag (A) or SNAPTag (B) fused IL12Rβ1, in the presence or absence of 50 nM unlabelled IL-23. Data is mean with SEM from five (A) or three (B) independent experiments. (C) The specific binding data shown in (A) and (B) with non-specific subtracted.

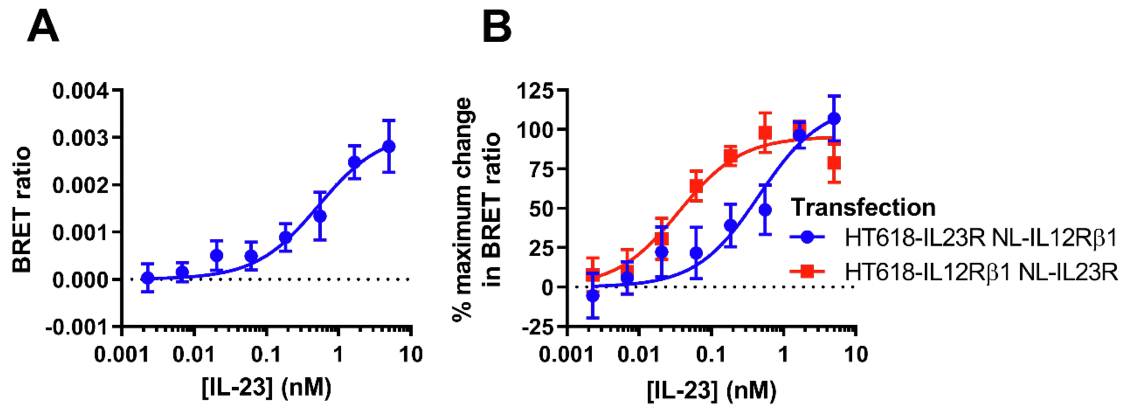

Figure S6 (Related to Figure 7): IL-23 mediated change in intra-receptor BRET measured using NL-IL12Rβ1 and HT618-IL23R. (A) The change in intra-receptor BRET between NL-IL12Rβ1 and HT618-IL23R expressed on HEK293T cells after incubation with unlabelled IL-23. Background normalised mean ± SEM BRET ratio values obtained from 4 independent experiments. (B) The data shown in (A) normalised to % change in BRET with equivalent data from cells transfected with HT618-IL12Rβ1 and NL-IL23R for reference.
